# Graph Neural Networks for D2D Link Scheduling: Centralized and Distributed Schedulers Evaluated with Sionna Ray-Traced Channels

**DOI:** 10.3390/s26206603

**Published:** 2026-10-18

**Authors:** Tae-Won Ban

**Affiliations:** Department of Artificial Intelligence and Information Engineering, Gyeongsang National University, Jinju 52828, Republic of Korea; twban35@gnu.ac.kr; Tel.: +82-55-772-9177

**Keywords:** device-to-device communication, link scheduling, interference management, graph neural networks, graph attention network, reinforcement learning, distributed resource allocation, ray tracing, network sensing, 6G networks

## Abstract

Device-to-device (D2D) communication requires a link scheduler that manages interference among simultaneously active pairs, an NP-hard problem even under binary on/off transmission. Learning-based schedulers are mostly trained and evaluated under stochastic channel models that miss site-specific propagation. We study learned D2D scheduling under Sionna ray-traced channels of downtown Munich. Two schedulers are trained with policy-gradient reinforcement learning to maximize the sum-rate: a centralized graph-attention scheduler that observes the full channel matrix, and a fully distributed per-pair policy that uses only locally measurable channel statistics, with no inter-pair message exchange at decision time. On 24,000 snapshots with N=10 pairs, the centralized scheduler reaches 94.6% of the brute-force-optimal sum-rate at 1.1% of its computation time on a GPU (25% on one CPU thread), rising to 97.5% with a local-search refinement, and, without retraining, stays within 94.4–95.6% of optimal across N=2–10, i.e., up to its training size. The distributed scheduler attains 70.7% of optimal (mean of five runs), quantifying the accuracy cost of decentralized execution, which grows with the number of pairs. Under independent channel estimation errors, both retain more than 97% of their accuracy, whereas missing estimates of silent peers affect the centralized scheduler more strongly.

## 1. Introduction

Device-to-device (D2D) communication, in which nearby mobile terminals exchange data directly instead of relaying traffic through a base station, has long been regarded as a key enabler for improving spectral efficiency, reducing latency, and offloading cellular infrastructure in 5G and beyond [1]. When D2D pairs reuse cellular spectrum in an underlay fashion, they generate mutual co-channel interference, so a scheduler is required to decide, slot by slot, which subset of pairs may transmit simultaneously. Even under the simplest binary activity model—each pair either transmits at a fixed power or stays silent—this decision is a non-convex combinatorial problem that is NP-hard in general [2]. It is closely related to, but not equivalent to, the maximum-weight independent-set (MWIS) problem on a pairwise conflict graph [3], because each link rate depends on the aggregate interference from all active pairs (Section 3.1), and it is solved exactly here only by an exhaustive search over $2^{N}$ candidate schedules; this combinatorial explosion motivates learning-based schedulers that can produce near-optimal decisions at a fraction of the cost of exhaustive or iterative optimization. Beyond conventional user-equipment traffic offloading, this direct-link scheduling problem is equally central to the infrastructure-free sensor and machine-type device networks envisioned for 6G, where battery- and bandwidth-constrained IoT sensor nodes must autonomously coordinate proximity-based links under tight latency and energy budgets [4]; the schedulers developed in this paper apply to such sensor-to-sensor D2D links, not only to conventional user–equipment pairs.

Indeed, a broad body of prior work already addresses link scheduling and power control in interference-limited wireless networks, spanning classical optimization-based algorithms, deep-learning and graph-neural-network (GNN) policies, distributed multi-agent reinforcement learning, and, more recently, ray-traced and digital-twin channel datasets. Section 2 reviews this literature and positions the present work relative to it (Table 1). In brief, that review identifies two gaps that motivate this paper: (i) essentially all learning-based schedulers for D2D and similar interference networks are trained and evaluated on stochastic, spatially unstructured channel models rather than on site-specific, ray-traced propagation data, so how well they transfer to geometry-consistent, real-world-like conditions remains unclear; and (ii) very few studies place a centralized policy and a distributed counterpart that exchanges no messages at decision time side by side on the same dataset to quantify, rather than merely argue qualitatively, the accuracy cost of decentralization.

This paper addresses these gaps with the following contributions:

- We generate a dataset of 24,000 independent D2D network snapshots (N=10 pairs each) using Sionna ray tracing on the full three-dimensional Munich scene, with devices sampled along realistic pedestrian waypoints under intra-pair distance constraints, so that both large-scale path loss/shadowing and small-scale multipath fading emerge from deterministic electromagnetic propagation rather than from a stochastic model.
- We design and train, with REINFORCE-based policy-gradient reinforcement learning that directly maximizes network sum-rate, a centralized graph-attention scheduler that observes the full channel matrix and a fully distributed scheduler that uses only the locally measurable row/column statistics available to each individual D2D pair, with no message passing between pairs at inference time (the row and column are acquired passively, by overhearing standard sidelink transmissions).
- We benchmark both schedulers against brute-force-optimal, FPLinQ, greedy, and threshold-based baselines—as well as against the graph-embedding scheduler of Lee et al., evaluated via the authors’ public implementation ported to our dataset—in terms of sum-rate, percentage of the optimal sum-rate, and per-sample inference latency, and we quantify the accuracy loss that is the price of fully decentralized execution.
- We evaluate the size generalization of both schedulers by testing them, without retraining, on networks with N=2 to 9 pairs after training only at N=10 (i.e., generalization to networks smaller than the training size; extrapolation to larger networks is not evaluated): the centralized scheduler stays within a band of less than two percentage points across the whole range, while the distributed scheduler’s accuracy declines monotonically as *N* grows, so the centralized–distributed gap widens with network size.
- We relax the perfect-CSI assumption by driving every scheduler with channel estimates corrupted under a power-preserving per-link error model while still evaluating the achieved sum-rate on the true channel, and show that the proposed schedulers retain more than 97% of their perfect-CSI accuracy under this model; we further evaluate silent peers with missing or stale estimates, SNR-dependent estimation error, and quantized reporting, which reveals a markedly higher sensitivity of the centralized scheduler to missing channel estimates.
- We verify these findings by retraining every learned scheduler with five independent random seeds, reporting seed-paired confidence intervals for the main margins, and by a feature ablation of the distributed scheduler, which shows that its accuracy saturates at about 71% of optimal once row and column statistics are combined, with rapidly diminishing returns from further local features.

The remainder of the paper is organized as follows. Section 2 reviews related work and positions this paper’s contributions relative to it. Section 3 formulates the D2D scheduling problem and describes the ray-traced channel model. Section 4 presents the proposed centralized and distributed schedulers and their training procedure. Section 5 describes the baseline algorithms and evaluation metrics and reports the resulting sum-rate, optimality gap, latency, size-generalization, imperfect-CSI robustness, and operating-point sensitivity results. Section 6 discusses the centralized–distributed trade-off and the limitations of the study, and Section 7 concludes the paper.

## 2. Related Work

### 2.1. Classical and Optimization-Based Link Scheduling and Power Control

Before learning-based approaches, D2D and, more generally, interference-network scheduling and power control were addressed with model-based optimization. The weighted minimum mean-square-error (WMMSE) algorithm reformulates weighted sum-rate maximization as a block-coordinate-descent problem and iterates to a stationary point given full channel state information (CSI) at a central point [5]; it remains a standard non-learning baseline [15] but requires tens of iterations for every channel realization. In the same centralized spirit but for the binary scheduling problem itself, FPLinQ reformulates joint link selection via fractional programming and iterates closed-form updates given full CSI [16], which are guaranteed to converge but, unlike exhaustive search, not to a global optimum; it also serves as the label generator for the learning-based scheduler of Lee et al. [11] discussed in Section 2.2, and both are evaluated quantitatively in Section 5. A second family dispenses with centralized optimization altogether and admits links according to a simple threshold rule. FlashLinQ established this approach with a synchronous distributed scheduler in which contending links yield to one another on the basis of locally measured signal-to-interference ratios, using an analog energy-level signaling scheme [17]. Naderializadeh and Avestimehr’s ITLinQ subsequently replaced FlashLinQ’s heuristic SIR rule with an information-theoretically motivated condition—scheduling links whose mutual interference-to-signal ratios stay below a threshold derived from the optimality conditions of treating interference as noise—implemented with a lightweight two-phase distributed signaling protocol, and reported substantial sum-rate gains over FlashLinQ at comparable complexity [6]. More recently, GRLinQ has combined the information-theoretic insights behind these rules with graph reinforcement learning, which relaxes their CSI requirements [18]. Binary scheduling with SINR-based rates is NP-hard in general [2]. When interference is abstracted into pairwise binary conflicts, it becomes an MWIS problem on the resulting conflict graph [3]; under the aggregate-interference model used in this paper, however, MWIS is a related abstraction rather than an exact reformulation (Section 3.1). Exact solutions such as brute-force search are used in this paper only as an offline reference upper bound (Section 5.1), not as a deployable algorithm. None of these classical methods amortizes its computation: WMMSE re-solves an optimization problem, and ITLinQ-style rules re-run their signaling protocol at every scheduling instant, in contrast to a trained neural network that pays a one-time training cost and then only a cheap forward pass at run time.

### 2.2. Deep Learning and Graph Neural Networks for Radio Resource Management

A first wave of learning-based work replaced iterative optimizers with feedforward approximators: Sun et al. showed that a fully connected deep neural network (DNN) can learn to imitate WMMSE’s input–output mapping directly, running orders of magnitude faster at inference [7], but such DNNs are tied to a fixed number of links. This motivated graph-structured architectures that treat the interference pattern itself as a graph. Eisen and Ribeiro’s random-edge GNN (REGNN) performs convolutions over the fading-induced interference graph and is provably permutation-equivariant, allowing transference across differently sized and topologically distinct networks [8]; Shen et al. formalized design principles and theoretical generalization guarantees for GNN-based radio resource management [9] and later surveyed the broader design space [19]. Chowdhury et al. hybridized the model-based and data-driven views by unrolling WMMSE’s iterations into a GNN with learned, graph-parameterized weights (UWMMSE) [10]. Graph attention networks [20], which let a model learn rather than hand-design the relative importance of each neighbor, underlie the message-passing layers of the centralized scheduler proposed in Section 4.1. Closest to the present setting, Lee, Yu, and Li proposed a graph-embedding-based method for binary D2D link scheduling that achieves near-optimal performance from only a few hundred labeled training samples [11]. However, that method (i) is supervised, whereas the schedulers trained here require no labels in their primary REINFORCE-based training; (ii) is evaluated under a stochastic, distance-and-shadowing-based channel model rather than site-specific ray-traced propagation; and (iii) is inherently centralized. Treating binary link scheduling as an MWIS problem on a conflict graph with given per-link utilities, Zhao et al. proposed graph-convolutional-network heuristics in both a centralized form—tree search guided by a GCN with one-step rollout—and a distributed form, in which a GCN produces topology-aware node embeddings that are combined with per-link utilities before a distributed greedy solver is invoked [3]. Of the studies reviewed here, this is the closest to the present work, in that it likewise places a centralized and a distributed learned scheduler side by side. It differs in two respects that matter for our purposes: it operates on abstract conflict graphs with given per-link utilities rather than on physical channel realizations, so no propagation model enters its evaluation at all; and its distributed variant still exchanges information with neighboring links at scheduling time, whereas the distributed scheduler proposed in Section 4.2 exchanges no messages at scheduling time. More broadly, the GNN-based RRM literature summarized above is evaluated either under stochastic channel models or, as in [3], on abstract graphs, leaving open how these architectures behave under the deterministic, geometry-consistent shadowing and multipath structure produced by real building layouts.

### 2.3. Distributed and Multi-Agent Reinforcement Learning for Wireless Resource Allocation

A parallel line of work asks whether a resource-allocation policy can be executed without a central controller collecting the full network state. Nasir and Guo trained distributed deep Q-learning agents for power control that act on delayed local and neighboring-cell CSI and interference measurements, exchanged among nearby transmitters at every decision instant [13]. Wang, Eisen, and Ribeiro’s decentralized GNN restricts message passing to a bounded *K*-hop neighborhood, but still requires each agent to exchange information with its *K*-hop neighbors at every scheduling instant [12]. Li and Guo proposed a D2D-specific multi-agent actor–critic scheme (NAAC) that uses neighboring pairs’ historical information only during centralized training and claims no signaling at execution [14], following the centralized-training/decentralized-execution (CTDE) paradigm popularized by MADDPG [21]; both NAAC and the present paper’s distributed scheduler (Section 4.2) adopt CTDE, but NAAC is evaluated on a stochastic channel model and does not report a matched comparison against a centralized counterpart trained and evaluated under identical conditions. With the exception of Nasir and Guo’s value-based deep Q-learning [13], the above training procedures [12,14,21]—as well as both schedulers proposed here—rely on some form of policy-gradient learning, the family introduced by Williams’ REINFORCE algorithm [22], which optimizes an expected reward (here, sum-rate) directly without requiring a differentiable system model. Relative to this literature, the distributed scheduler developed in this paper is more strictly decentralized: it uses zero-hop information (each pair’s own row/column of the channel matrix only, with no exchange with any other pair at inference time, although acquiring them relies on overhearing the other pairs’ standard transmissions, cf. Section 4.2), rather than the one- or multi-hop neighbor exchange used in [12,13], and it is trained with a simple sample-based (mean or leave-one-out) policy-gradient baseline (Section 4.3) rather than a learned critic network.

### 2.4. Ray-Tracing and Site-Specific Channel Modeling for Learning-Based Wireless Systems

Differentiable, GPU-accelerated ray tracers such as Sionna RT [23,24] and parameterized ray-traced datasets such as DeepMIMO [25] now make it practical to generate large volumes of site-specific channel realizations from real or synthetic three-dimensional scenes, and such data is increasingly used to close the simulation-to-deployment gap for network-level machine learning and digital-twin studies targeting 6G network simulation [26]. To date, however, these tools have been applied predominantly to tasks such as beam prediction, localization, and channel estimation; ray-traced data has rarely been used to train resource-scheduling policies with reinforcement learning, and, to the best of our knowledge, no prior work has trained and compared centralized and fully distributed D2D schedulers side by side on a shared ray-traced dataset. The closest such comparison [3] is conducted on abstract conflict graphs rather than on physical channel realizations, so it does not speak to how site-specific propagation affects the centralized–distributed trade-off. This paper’s dataset (Section 3.2) and evaluation protocol are designed to fill exactly this gap.

### 2.5. Positioning of This Work Relative to Prior Studies

Table 1 compares this work with the eleven representative studies reviewed above in terms of the channel model, the decision variable, the execution mode, the training paradigm, and the information exchanged between links at run time. Read column by column, it shows both where the present study goes beyond most of these studies and where it does not.

The present study goes beyond most of the studies in Table 1 in three respects. First, it is the only one whose schedulers are trained and evaluated on site-specific, ray-traced channels; all of the other learning-based and rule-based schemes were developed and evaluated under stochastic channel models or, in the case of Zhao et al. [3], on abstract conflict graphs with no propagation model at all, and WMMSE [5] is an optimization algorithm that applies to any given CSI. Second, apart from Zhao et al., it is the only study that places a centralized and a distributed learned scheduler side by side, and it does so with both schedulers trained on the same reward and dataset and evaluated on the same test set, so that the accuracy cost of decentralization is measured rather than argued. Third, its distributed variant requires no message exchange between pairs at decision time (its inputs are acquired by passively overhearing the other pairs’ transmissions), a stricter requirement than the per-slot signaling of ITLinQ [6], the neighbor CSI exchange of Nasir and Guo [13], the *K*-hop exchange of Wang et al. [12], and the neighbor exchange of the distributed variant of Zhao et al. [3]; only NAAC [14] claims the same, under a stochastic channel model and without a matched centralized counterpart. The label-free training of both schedulers, by contrast, is shared with most of the learning-based studies in the table; it distinguishes this work only from the supervised approaches of Sun et al. [7] and Lee et al. [11], whose accuracy is bounded by that of the teacher algorithm that produces their labels. Among the learning-based studies in the table, only the scheduler of Lee et al. addresses the same binary problem with information that is available in our setting and could therefore be evaluated on our dataset; there, it attains 42.3% of the optimal sum-rate, against 94.6% and 70.7% for the proposed centralized and distributed schedulers (Section 5.3), a comparison that is specific to this setting.

The table equally shows the limitations of the present study. First, eight of the eleven studies optimize a continuous or multi-level transmit power, a beamformer, or a spectrum assignment, which is a strictly larger action space than the binary on/off decision considered here; the proposed schedulers cannot exploit partial power reduction and therefore address a more restricted problem. Second, the ray-traced evaluation covers a single urban scene, a single carrier frequency, and single-antenna devices, whereas the stochastic models used in prior work, although less realistic, can be varied easily over wide ranges of densities and topologies. Third, both schedulers are trained at N=10 and evaluated only for N≤10, whereas several GNN-based studies report transfer to networks larger than those seen in training [8,10,12]. Fourth, by exchanging no messages at all, the distributed variant forgoes the neighbor information that the signaling-based distributed methods exploit, and it should be expected to be less accurate than such methods when they are given that additional information budget. Finally, because the studies in Table 1 use different channel models, decision variables, and network sizes, their reported performance figures are not directly comparable with ours; a quantitative comparison is therefore restricted to the methods that can be evaluated on the same dataset under the same binary formulation (Section 5.1).

## 3. System Model

### 3.1. D2D Link Scheduling Problem

**Figure 1:**
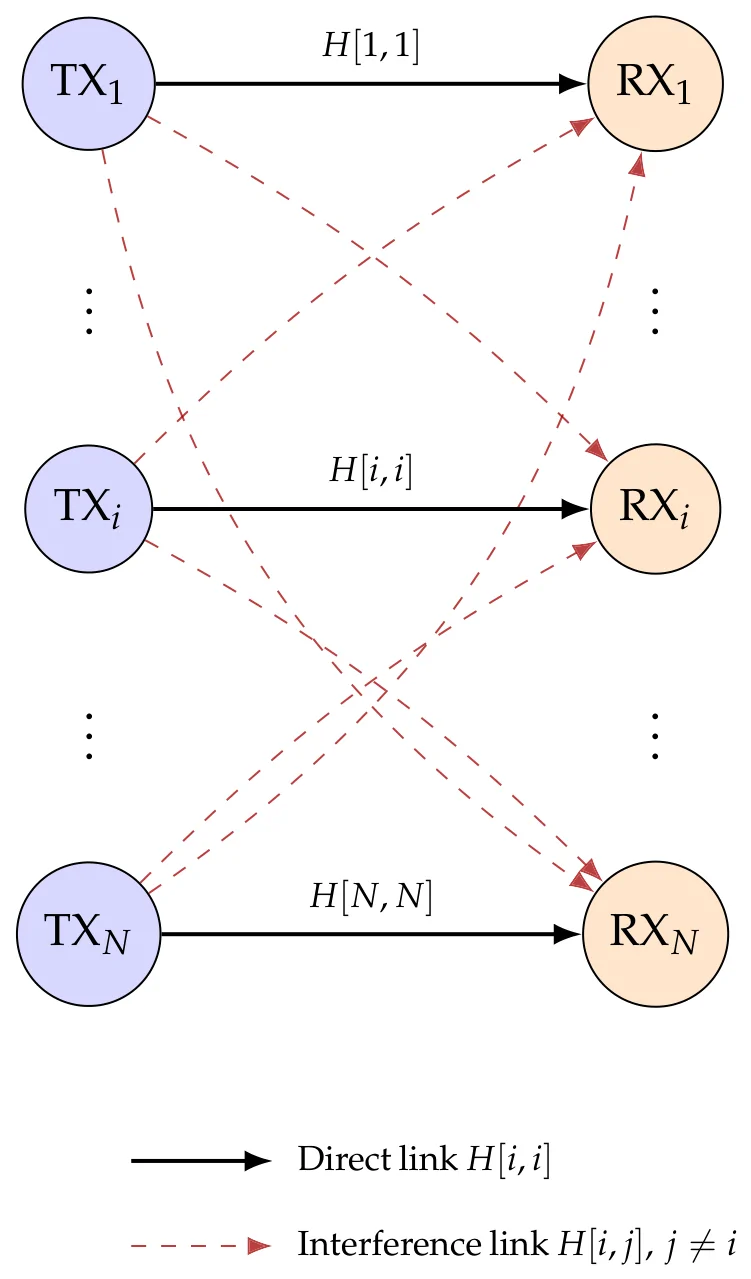
Illustrative D2D scheduling scenario with *N* co-channel pairs, showing pairs 1, *i*, and *N* as representatives; the omitted pairs 2,…,i−1 and i+1,…,N−1 are indicated by vertical ellipses. Solid arrows denote desired direct links H[i,i]; dashed arrows denote cross-link interference channels H[i,j], j≠i (a representative subset is shown, unlabeled, to keep the diagram readable). The scheduler decides the binary activity vector $m\in {\left\{0,1\right\}}^{N}$ that maximizes the network sum-rate given the full interference pattern.

We consider a D2D network consisting of *N* co-channel transmitter–receiver pairs sharing a common spectrum, as illustrated in Figure 1. Each pair i∈N≜{1,2,…,N} comprises a transmitter ${\mathrm{TX}}_{i}$ and an intended receiver ${\mathrm{RX}}_{i}$, and each device is equipped with a single antenna. Let $H\in C^{N\times N}$ denote the narrowband channel matrix of the network, where the entry H[i,j] represents the complex baseband channel coefficient from ${\mathrm{TX}}_{j}$ to ${\mathrm{RX}}_{i}$. The diagonal entries ${\left\{H\left[i,i\right]\right\}}_{i\in N}$ correspond to the desired (direct) links, while the off-diagonal entries represent cross-link interference channels.

In each scheduling slot, a binary scheduling vector

(1) $$m={\left[m_{1},m_{2},\ldots ,m_{N}\right]}^{T}\in {\left\{0,1\right\}}^{N}$$

is determined, where $m_{i}=1$ indicates that pair *i* transmits in the current slot, and $m_{i}=0$ indicates that pair *i* remains silent. Each active transmitter emits at the same transmit power $P_{\mathrm{tx}}$ (linear scale), and additive white Gaussian noise with power $P_{n}$ is observed at every receiver.

**Received power and interference.** The signal model in (2)–(5) is the standard narrowband interference-channel model with single-antenna nodes, in which interference is treated as noise [27]; the same model underlies the binary link-scheduling formulations of [6,16]. When ${\mathrm{TX}}_{j}$ transmits, the received power at ${\mathrm{RX}}_{i}$ from ${\mathrm{TX}}_{j}$ is

(2) $$P_{\mathrm{rx}}\left[i,j\right]\;=\;P_{\mathrm{tx}}\;{\left|H\left[i,j\right]\right|}^{2}.$$

The desired signal power at ${\mathrm{RX}}_{i}$ when pair *i* is active is $m_{i}\;P_{\mathrm{rx}}\left[i,i\right]$, and the aggregate co-channel interference from all other active pairs is

(3) $$I_{i}\left(m\right)\;=\;\sum_{j\in N,\;j\neq i}m_{j}\;P_{\mathrm{rx}}\left[i,j\right].$$

**SINR and link rate.** The signal-to-interference-plus-noise ratio (SINR) of pair *i* under scheduling vector m is

(4) $$\gamma_{i}\left(m\right)\;=\;\frac{m_{i}\;P_{\mathrm{rx}}\left[i,i\right]}{I_{i}\left(m\right)+P_{n}}.$$

By the Shannon formula [27], the achievable instantaneous spectral efficiency of pair *i* is $\log_{2}\left(1+\gamma_{i}\left(m\right)\right)$ bps/Hz; if $m_{i}=0$ then $\gamma_{i}=0$ and the corresponding rate vanishes.

**Sum-rate and scheduling objective.** The network sum-rate associated with scheduling vector m is

(5) $$R_{\mathrm{sum}}\left(m\right)\;=\;\sum_{i=1}^{N}\log_{2}\;\left(1+\gamma_{i}\left(m\right)\right).$$

The objective of D2D link scheduling considered in this work, as in [6,16], is to find the binary scheduling vector that maximizes the network sum-rate for a given channel realization H:

(6) $$m^{★}\left(H\right)\;=\;\underset{m\in {\left\{0,1\right\}}^{N}}{arg max}\;R_{\mathrm{sum}}\left(m\right).$$

Problem (6) is a non-convex combinatorial optimization with a feasible set of cardinality $2^{N}$. It is NP-hard in general: in the special case of equal direct-link gains and cross-link gains that are either zero or so large that two mutually interfering pairs are never worth activating together, (6) reduces to a maximum independent-set problem on the graph of interfering pairs, which is the reduction underlying the strong NP-hardness of sum-rate maximization in interference networks established in [2]. For general channels, however, (6) is *not* equivalent to a maximum-weight independent-set (MWIS) problem: through (4), the rate of each active pair depends on the aggregate interference from all other active pairs, so the objective cannot be written as a sum of fixed per-pair weights subject to pairwise exclusion constraints. MWIS on a conflict graph, obtained by thresholding the cross-link gains into binary conflicts and assigning each pair a fixed utility, is therefore a related abstraction that underlies graph-based scheduling heuristics [3], not an exact reformulation of (6). Exhaustive search solves it exactly but at a cost that grows exponentially with *N*, motivating the low-complexity centralized and fully distributed learning-based schedulers developed in Section 4.1 and Section 4.2.

### 3.2. Ray-Traced Channel Model

To capture realistic propagation effects in an urban D2D deployment, we adopt a ray-tracing-based channel model rather than a stochastic abstraction (e.g., i.i.d. Rayleigh fading). The channel coefficients {H[i,j]} used throughout this paper are obtained from deterministic electromagnetic propagation simulation on a real-world three-dimensional scene, as detailed below.

#### 3.2.1. Propagation Environment and Channel Computation

We employ the Sionna ray tracer (Sionna RT) [23,28,29] together with the publicly available Munich scene, which contains a detailed 3D geometric and material representation of a section of downtown Munich. The carrier frequency is $f_{c}=3$ GHz, and all devices operate at the same height h=1.5 m above ground, consistent with pedestrian-level D2D deployment. Each transmitter and receiver is equipped with a single vertically polarized short-dipole antenna, modeled in Sionna RT as a 1×1 PlanarArray with the built-in dipole pattern, whose directivity is 1.5 (1.76 dBi) in the horizontal plane.

For each network realization, the propagation paths between every transmitter and every receiver are computed by tracing electromagnetic rays within the scene, with a maximum interaction depth of $L_{\max}=5$ per ray. The model accounts for line-of-sight (LoS) paths, specular reflections off buildings and the ground, and refraction (transmission) through building surfaces, so non-line-of-sight (NLoS) links are served by multi-bounce specular and transmitted paths. Because the paths are computed from the actual 3D geometry, path loss, building-induced shadowing, and multipath fading all emerge from the simulation rather than being modeled as separate stochastic terms.

For each ordered index pair (i,j)—receiver ${\mathrm{RX}}_{i}$ and transmitter ${\mathrm{TX}}_{j}$, following the convention used for H—the ray tracer returns a set of $L_{ij}$ resolved propagation paths characterized by complex amplitudes ${\left\{a_{\ell }\left(i,j\right)\right\}}_{\ell =1}^{L_{ij}}$ and propagation delays ${\left\{\tau_{\ell }\left(i,j\right)\right\}}_{\ell =1}^{L_{ij}}$. Assuming narrowband transmission centered at $f_{c}$, the equivalent baseband channel coefficient is obtained by coherently summing the path contributions with their carrier-induced phase rotations [27,28]:

(7) $$H\left[i,j\right]\;=\;\sum_{\ell =1}^{L_{ij}}a_{\ell }\left(i,j\right)\;\exp\;\left(-j\;2\pi f_{c}\;\tau_{\ell }\left(i,j\right)\right).$$

The amplitudes $\left\{a_{\ell }\right\}$ encode path-specific attenuation from free-space propagation, reflection and transmission losses at building surfaces, and antenna patterns, so (7) implicitly captures distance- and obstacle-dependent path loss and shadowing, while multipath fading arises from the coherent sum of contributions with different delays.

Because the antenna patterns are already contained in the path amplitudes $a_{\ell }$, and hence in H, no separate antenna gain is added to the link budget: the transmit power in (2) is the conducted power per transmitter, $P_{\mathrm{tx}}^{\left(\mathrm{dBm}\right)}$ (Table 2), converted to watts as

(8) $$P_{\mathrm{tx}}\;=\;10^{\left(P_{\mathrm{tx}}^{\left(\mathrm{dBm}\right)}-30\right)/10}.$$

The short-dipole pattern contained in H is therefore the only antenna gain in the model: no further antenna gain is applied at the transmitter or at the receiver, and the conducted transmit power is not increased by any antenna gain.

#### 3.2.2. Device Placement and Dataset Generation

For each network realization, the locations of the 2N devices are drawn from candidate positions defined along waypoint polylines that lie on traversable streets and sidewalks within the Munich scene, so devices are always placed in physically meaningful outdoor locations. The transmitter ${\mathrm{TX}}_{i}$ of pair *i* is sampled uniformly along the polylines. To reflect the short-range nature of D2D communication, the corresponding receiver ${\mathrm{RX}}_{i}$ is sampled independently along the polylines subject to the upper bound of the constraint

(9) $$d_{\min}\;\leq \;{∥x_{{\mathrm{TX}}_{i}}-x_{{\mathrm{RX}}_{i}}∥}_{2}\;\leq \;d_{\max},$$

where $x_{{\mathrm{TX}}_{i}},x_{{\mathrm{RX}}_{i}}\in R^{3}$ are the 3D positions of the transmitter and receiver of pair *i*, $d_{\max}=50$ m is the maximum allowed intra-pair distance, and $d_{\min}=1$ m is the minimum. The upper bound, enforced during sampling, reflects the short-range nature of D2D communication; the lower bound is enforced afterward by discarding the snapshots that violate it (see below) and is imposed for two reasons. First, at $f_{c}=3$ GHz the wavelength is λ=9.99 cm, so $d_{\min}=10\lambda$ keeps every intra-pair link comfortably in the far field of the dipole antennas, where the ray-optical propagation model used by the ray tracer is valid. Second, a transmitter–receiver separation below one meter would in practice correspond to two devices carried by the same user, which is not a meaningful D2D scheduling instance. The constraint applies within a pair only: different pairs share the same scene and may be located arbitrarily close to one another, giving rise to the cross-link interference that the scheduler must resolve. Candidate placements without at least one resolved propagation path between ${\mathrm{TX}}_{i}$ and ${\mathrm{RX}}_{i}$ are discarded and re-sampled.

Apart from this near-field exclusion, the ray-traced propagation itself requires no correction: across all distance bins, the median direct-link gain ${\left|H\left[i,i\right]\right|}^{2}$ stays within a small factor of the free-space value ${\left(\lambda /4\pi d\right)}^{2}$ (between 1.1 and 3.2 across 10 m distance bins, with an overall median of 2.4). This factor is accounted for by the gain of the two dipole antennas embedded in the path coefficients, $1.5^{2}=2.25$ (3.5 dB): for links shorter than 5 m, where the line-of-sight path dominates, the median factor is 2.28, while the spread across distance bins reflects ground and building reflections and shadowing.

**Figure 2:**
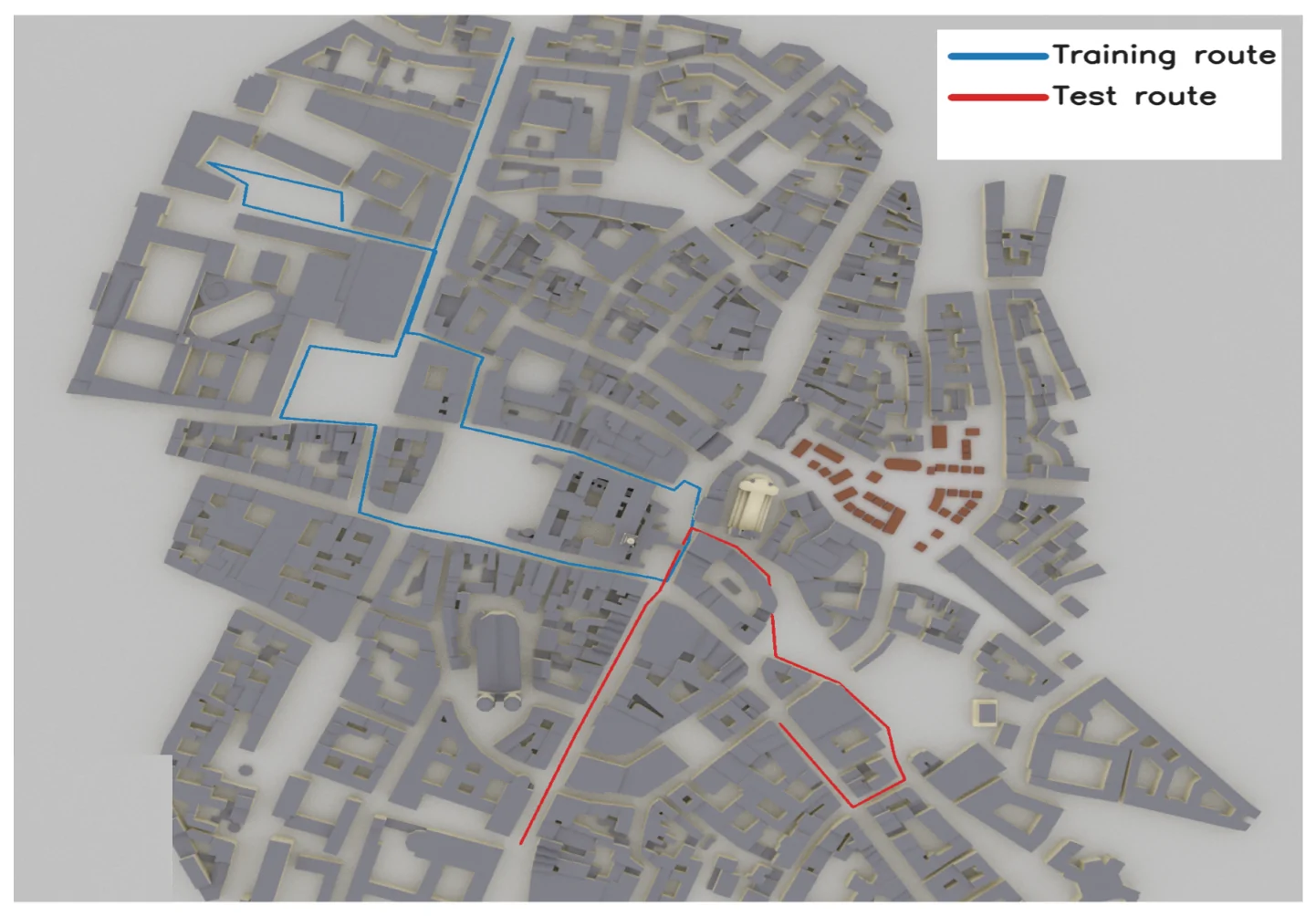
Top-down view of the Sionna Munich ray-traced scene with the pedestrian waypoint routes overlaid. The training route (blue) and test route (red) mark the polylines along which transmitter and receiver positions are sampled for D2D pair placement. The two routes occupy disjoint regions of the scene.

Figure 2 shows the top-view layout of the two disjoint pedestrian polylines used to sample device positions. The training route (2127 m, 30 waypoints) and the test route (993 m, 15 waypoints) lie in spatially separate parts of the Munich scene, so the geometric context available at test time is never seen during training—a stronger generalization test than a random split of positions along a shared route.

We fix N=10 pairs per snapshot and generate 30,000 independent snapshots in total. Snapshots in which any of the ten pairs violates the minimum-distance bound of (9) are discarded, and the survivors are then truncated to round counts, giving the 24,000 snapshots used in this paper: 16,000 training snapshots and 8000 evaluation snapshots, the latter further split into 2000 validation and 6000 test snapshots. Because the validation and test snapshots originate from the same test route, they are pooled after filtering and re-split, while the training snapshots remain drawn from their own disjoint route, so training and evaluation geometries never overlap. All snapshots are statistically independent: positions are re-sampled independently and uniformly at random along the waypoint polylines, rather than obtained by advancing along a continuous, speed-constrained pedestrian trajectory, so no temporal correlation is modeled. The complete set of simulation parameters is summarized in Table 2.

## 4. Proposed Schemes

### 4.1. Proposed Centralized Graph-Attention Scheduler

The centralized scheduler treats the *N* D2D pairs as nodes of a fully connected graph and H as the corresponding edge weights, and it outputs a scheduling decision for all pairs jointly. Each node *i* is initialized with a two-dimensional feature $\left[\;g_{i},\;c_{i}\;\right]$. All channel gains are first mapped to a fixed affine dB scale, $\tilde{G}\left[i,j\right]=\left(10\log_{10}{\left(|H\left[i,j\right]\right|}^{2}+\delta )+80\right)/40$, where $\delta =10^{-15}$ keeps entries without a resolved propagation path finite; this scale does not depend on the transmit or noise power. The node features are the normalized direct-link gain $g_{i}=\tilde{G}\left[i,i\right]$ and $c_{i}=\sum_{j\neq i}\tilde{G}\left[i,j\right]$, the sum of the normalized gains of the cross-links incoming to ${\mathrm{RX}}_{i}$, which serves as a log-domain summary of the interference environment at that receiver; each directed edge (j→i) is initialized with the one-dimensional feature $\tilde{G}\left[i,j\right]$. Node and edge features are first mapped to a common hidden dimension by linear encoders with a ReLU nonlinearity.

The network then applies L=3 graph-attention message-passing layers [20]. At layer *ℓ*, for every ordered pair (i,j) with j≠i, an attention logit is computed from the concatenation of the receiver, sender, and edge embeddings, following the graph-attention mechanism of [20] extended here with the edge embedding $e_{ij}$,

(10) $$a_{ij}^{\left(\ell \right)}=\mathrm{LeakyReLU}\;\left(w_{a}^{(\ell )⊤}\left[\;h_{i}^{\left(\ell -1\right)}\;∥\;h_{j}^{\left(\ell -1\right)}\;∥\;e_{ij}\;\right]\right),$$

and normalized over the incoming neighbors of *i* with a masked softmax, $\alpha_{ij}^{\left(\ell \right)}={\mathrm{softmax}}_{j\neq i}\left(a_{ij}^{\left(\ell \right)}\right)$. A message $\mu_{ij}^{\left(\ell \right)}$ is computed from the same concatenated input via a small MLP, aggregated as $\mu_{i}^{\left(\ell \right)}=\sum_{j\neq i}\alpha_{ij}^{\left(\ell \right)}\;\mu_{ij}^{\left(\ell \right)}$, and combined with the previous node state through a second MLP to produce $h_{i}^{\left(\ell \right)}$. After *L* layers, a two-layer readout MLP maps $h_{i}^{\left(L\right)}$ to a scalar logit, and a sigmoid converts the logit to an activation probability $p_{i}\in \left(0,1\right)$. At inference time, the scheduler outputs the hard decision $m_{i}=1\left\{p_{i}>0.5\right\}$ for every pair simultaneously; during training, the soft probability $p_{i}$ is used to sample stochastic actions (Section 4.3). The resulting model has 64,836 trainable parameters (hidden dimension 64). Because every layer computes an attention logit and a message for each of the N(N−1) ordered pairs, one forward pass requires $O(LN^{2}d^{2})$ arithmetic operations for hidden dimension *d*, i.e., it grows quadratically with the number of pairs (about 8.0, 31, 190, and 755 MFLOPs per network for N=10, 20, 50, and 100).

**Figure 3:**
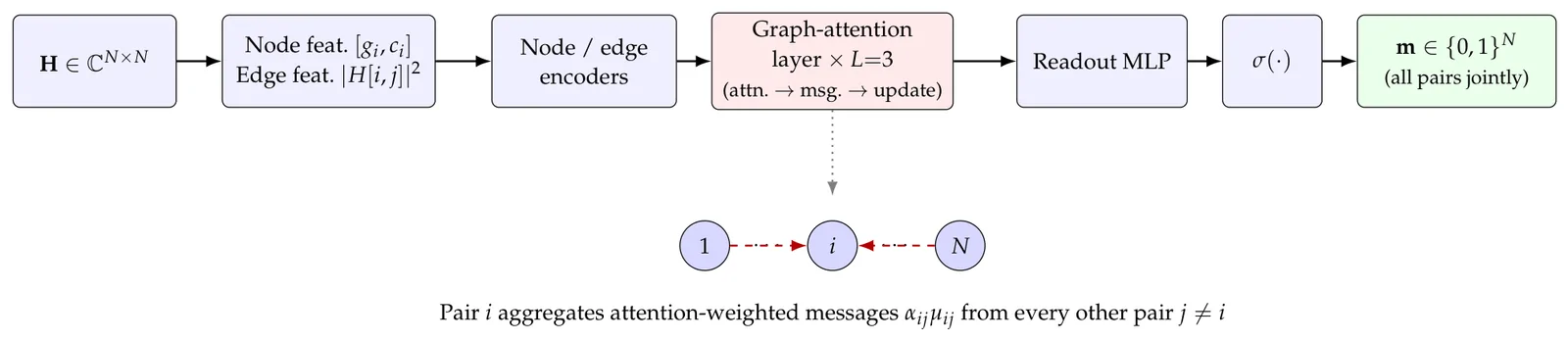
Conceptual architecture of the proposed centralized graph-attention scheduler. The full channel matrix H is encoded into per-pair node features and per-link edge features, refined through L=3 graph-attention message-passing layers in which every pair exchanges information with every other pair (inset), and mapped by a shared readout MLP and sigmoid to a joint scheduling decision m for all *N* pairs.

Figure 3 summarizes this pipeline. The key architectural property is that, at every layer, pair *i*’s representation is updated using attention-weighted messages aggregated from *every other pair*j≠i, so a scheduling decision for pair *i* can depend on information originating from any pair in the network; this full connectivity is what requires the complete matrix H to be available at a central point.

### 4.2. Proposed Distributed Scheduler

The distributed scheduler removes all message passing between pairs at inference time: pair *i* decides $m_{i}$ using only quantities that pair *i* itself can plausibly measure on a single-carrier time-division-duplex (TDD) sidelink, where channel reciprocity holds. Its own row H[i,:] of the channel matrix is estimated by ${\mathrm{RX}}_{i}$ from the demodulation and channel-state-information reference signals (DM-RS, sidelink CSI-RS) embedded in every sidelink transmission that it naturally overhears from each active transmitter [30]. Its own column H[:,i] is acquired by ${\mathrm{TX}}_{i}$ in the same passive, overhearing fashion from the receiver-side transmissions that the 3GPP sidelink specifications already mandate for unicast links—hybrid-ARQ feedback on the physical sidelink feedback channel (PSFCH) and reverse-link CSI reports [30,31]—each carrying a known sequence from which ${\mathrm{TX}}_{i}$ estimates the ${\mathrm{RX}}_{j}\to {\mathrm{TX}}_{i}$ channel and obtains H[j,i] by TDD reciprocity. Acquiring either vector therefore requires no pair-specific signaling protocol or explicit inter-pair feedback beyond procedures already standardized for the sidelink; the impact of the resulting estimation imperfection is quantified in Section 5.7. Throughout this paper, *fully distributed* refers to the decision stage: at scheduling time, each pair decides from its own locally acquired row and column of H, without exchanging any message with, or receiving any coordination signal from, another pair. It does not mean that these inputs are obtained without any over-the-air interaction: they are acquired passively from reference signals and feedback that the other pairs transmit for their own links, so their availability and freshness depend on how recently each peer has transmitted. Entries belonging to peers that have remained silent during the estimation window are stale or missing rather than merely noisy; this case is evaluated in Section 5.7.2. From these two vectors, we extract a 19-dimensional local feature per pair: the direct gain ${\left|H\left[i,i\right]\right|}^{2}$ (on the normalized dB scale $\tilde{G}$ of Section 4.1), and, separately for the row (received-interference side) and the column (transmitted-interference side), nine statistics each—the maximum, mean, standard deviation, median, and second-largest cross-link gain (dB); the log-SIR of the direct link against the total cross-link power and against the single strongest cross-link; the fraction of cross-links stronger than the direct link; and the direct link’s share of total received/caused power. This feature vector is passed through a weight-shared four-layer MLP (hidden dimension 128, ReLU) followed by a linear readout and a learnable scalar logit-scale *s* (initialized to 3.0) that sharpens the sigmoid output, $p_{i}=\sigma \left(s\cdot w^{⊤}h_{i}\right)$. Because the same weights are applied independently to every pair’s local feature vector, the policy is permutation-equivariant. The resulting model has 52,226 trainable parameters, 80.6% as many as the centralized model (about 204 KiB in 32-bit floating point, against 253 KiB for the centralized model). Each pair evaluates the MLP once on its own feature vector, which costs about 0.10 MFLOPs regardless of *N*; only the feature extraction, which reads the 2(N−1) cross-link gains of the pair, grows linearly with *N*.

**Figure 4:**
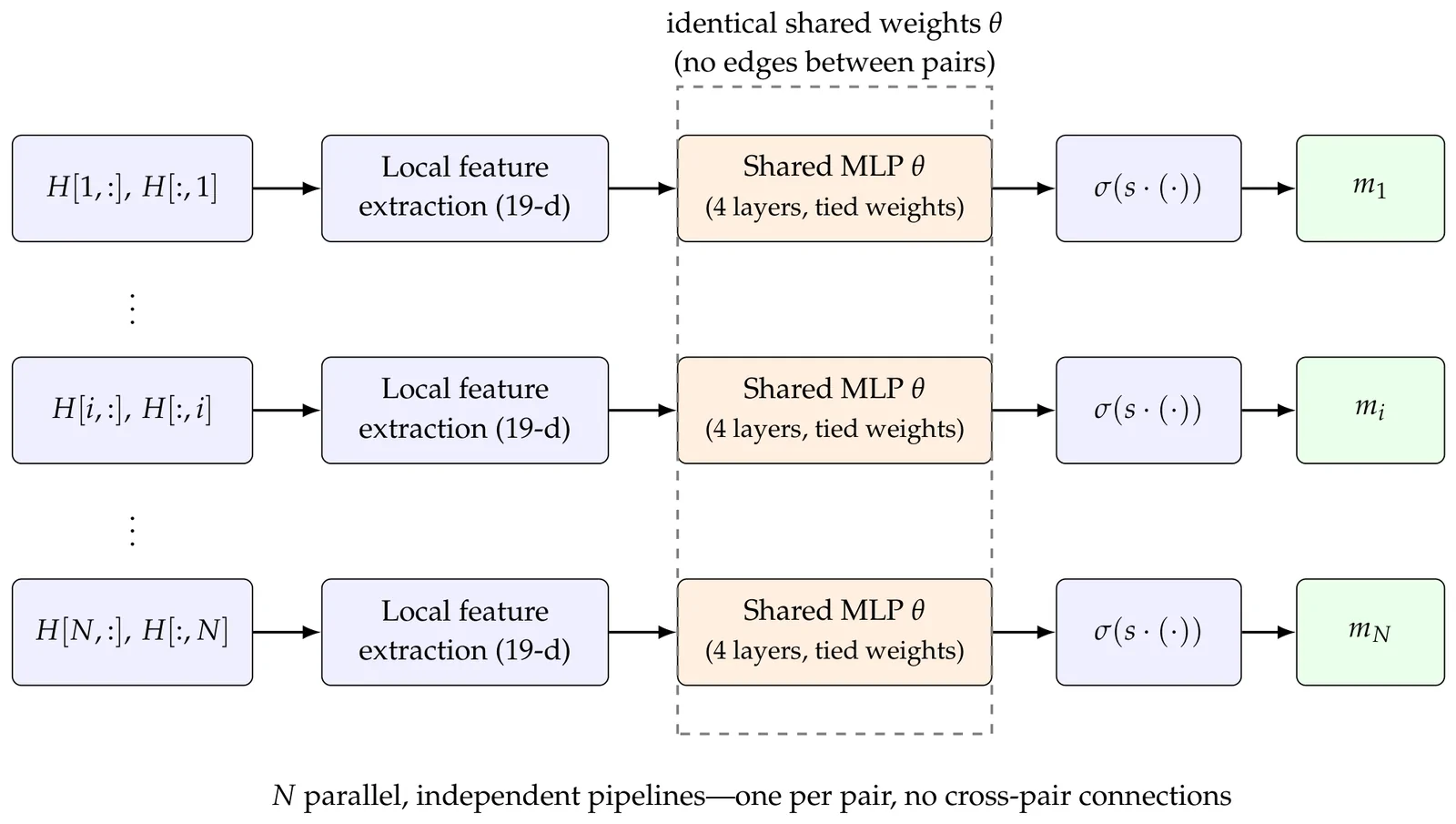
Conceptual architecture of the proposed distributed scheduler. Each pair *i* independently extracts a 19-dimensional local feature vector from its own row H[i,:] and column H[:,i] of H, and passes it through the same weight-shared MLP θ (dashed box) to obtain its own decision $m_{i}$. Unlike the centralized architecture of Figure 3, there is no message passing between the *N* parallel copies.

In contrast to the centralized scheduler of Section 4.1, this policy contains *no graph-attention or message-passing component*: it is not a graph neural network in the sense of exchanging information along edges, but a per-pair, weight-shared multilayer perceptron applied independently and identically to every pair’s own local feature vector, with no computational path connecting one pair’s decision to another pair’s input (Figure 4).

It is important to distinguish *weight sharing* from *distributed execution*, since the two describe different stages of the pipeline. Weight sharing means that a single parameter set θ is used by every pair rather than training *N* separate networks; this is purely a training-time and storage choice, adopted because it lets one policy be learned jointly from all pairs in every training network and because it requires distributing only a single small model to every device. It does not imply any run-time dependency between pairs: once training is complete, θ is a fixed set of numbers that is copied to every device once, in the same way that all users of a cellular network run identical receiver firmware. At execution time, pair *i* (i) locally forms its own 19-dimensional feature vector from H[i,:] and H[:,i], (ii) evaluates its own on-device copy of the trained MLP in a single feed-forward pass, and (iii) outputs $m_{i}$—with no message exchanged with, or information required from, any other pair during this process. Training itself follows the centralized-training/decentralized-execution (CTDE) paradigm (Section 4.3): the shared weights are learned offline from centrally available data, but the resulting policy is executed fully independently by each pair, so the scheme is genuinely distributed at run time despite the parameters being shared.

### 4.3. Training Procedure

Both schedulers are trained end-to-end with policy-gradient reinforcement learning, treating $R_{\mathrm{sum}}\left(m\right)$ from (5) directly as the reward, so that no labeled optimal schedule is required [22,32]. The network’s sigmoid outputs $\left\{p_{i}\right\}$ parameterize *N* independent Bernoulli random variables; S=8 scheduling vectors ${\left\{m^{\left(s\right)}\right\}}_{s=1}^{S}$ are sampled per channel realization, and the policy-gradient loss for the centralized model is

(11) $$L=-\frac{1}{S}\sum_{s=1}^{S}\left(R^{\left(s\right)}-b\right)\;\log\pi \left(m^{\left(s\right)}|H\right),\;b=\frac{1}{S}\sum_{s=1}^{S}R^{\left(s\right)},$$

where $R^{\left(s\right)}=R_{\mathrm{sum}}\left(m^{\left(s\right)}\right)$, $\log\pi \left(m|H\right)=\sum_{i}\left[m_{i}\log p_{i}+\left(1-m_{i}\right)\log\left(1-p_{i}\right)\right]$, and *b* is a sample-mean baseline computed from the *S* sampled schedules of the same channel realization that reduces gradient variance [22,33]. The distributed scheduler is trained with the same objective but with a leave-one-out baseline, $b^{\left(s\right)}=\frac{1}{S-1}\sum_{s^{\prime }\neq s}R^{\left(s^{\prime }\right)}$, which excludes each sample from its own baseline; since $R^{\left(s\right)}-b^{\left(s\right)}=\frac{S}{S-1}\left(R^{\left(s\right)}-b\right)$, it differs from the sample-mean baseline only by a constant factor, but it makes the policy-gradient estimate unbiased [33]. Both models are trained with Adam and a cosine-annealed learning rate; the centralized model uses batch size 128, learning rate $5\times 10^{-4}$, and 200 epochs, and the distributed model uses batch size 64, learning rate $5\times 10^{-4}$, and 300 epochs, with gradient norms clipped to 1.0. The checkpoint with the highest hard-threshold sum-rate on the validation set is kept for evaluation. Every learned scheduler is trained five times with independent random seeds (0–4), which fix the parameter initialization, the order of the training mini-batches, and the sampled actions; unless stated otherwise, reported accuracies are the mean over the five resulting models. We additionally implemented an imitation-pretrained variant of the distributed scheduler—following the same CTDE training/execution split as the REINFORCE-only model above—in which the policy is first pre-trained to imitate brute-force-optimal labels (computed at the operating point of Table 2) with a binary cross-entropy loss before REINFORCE fine-tuning; its evaluation against the REINFORCE-only distributed scheduler is reported in Section 6.

## 5. Results

### 5.1. Baseline Algorithms

We compare the proposed schedulers against the following baselines, grouped by the information they require.

*Centralized baselines* (require the full matrix H): (i) **Brute-force** exhaustively evaluates all $2^{N}$ candidate vectors and returns the optimal $m^{★}$, serving as the reference upper bound; (ii) **Greedy** activates pairs one at a time in decreasing order of direct-link gain ${\left|H\left[i,i\right]\right|}^{2}$, accepting each activation only if it increases the running sum-rate; (iii) **Local search (LS)** is a 1-bit-flip refinement applied on top of a given schedule (Greedy or the proposed centralized GNN): each pair’s activity bit is tentatively flipped and the flip is kept if it improves $R_{\mathrm{sum}}$, iterated in sweeps over all *N* pairs until no flip improves the schedule or *N* sweeps are reached; because it recomputes the exact sum-rate after every flip, LS also requires centralized knowledge of H.

*Distributed baselines* (use only pair-local information, matching the information available to the proposed distributed scheduler): (iv) **Direct threshold** activates pair *i* iff its own SNR $P_{\mathrm{tx}}{\left|H\left[i,i\right]\right|}^{2}/P_{n}$ exceeds 0 dB, ignoring interference entirely; (v) **SIR-local** activates pair *i* iff its direct gain exceeds its single strongest measured cross-link gain by at least 3 dB; (vi) **All-on** activates every pair unconditionally, representing the absence of any coordination. The threshold rules of Section 2.1—FlashLinQ [17] and ITLinQ [6]—are deliberately excluded from this group: both resolve contention by having lower-priority links yield to already admitted ones, which requires per-slot signaling between links, whereas every method grouped here, including the proposed distributed scheduler, decides from pair-local measurements with no message exchange at decision time. They therefore operate under a strictly larger information budget, and including them would compare different information assumptions rather than different policies.

*Reference-only baseline*: (vii) **Highest-one** activates only the single pair with the largest direct gain, representing an extremely conservative, interference-free operating point.

Two methods from the literature reviewed in Section 2 are additionally included in Table 3. FPLinQ [16] is a non-learned fractional-programming scheduler that addresses the same binary problem (6) given full CSI and requires no tuning; its closed-form iterations are guaranteed to converge, but only to a stationary point of a continuous relaxation that must then be binarized, so, unlike brute-force search, which is the only exact reference in this paper, it carries no global-optimality guarantee; we run its closed-form iterations to a fixed budget of 1000 (its accuracy plateaus well before this) and binarize with the rounding rule of its public reference implementation, reporting it both alone and with the same local-search refinement applied to the other centralized methods. The graph-embedding scheduler of Lee et al. [11] is the one learning-based method that shares our decision variable and operates on physical channel realizations; we evaluate it by porting the authors’ public implementation—architecture, quantized-distance features, structure2vec hyperparameters, FPLinQ training labels, and the few-hundred-sample training protocol unchanged—to the ray-traced dataset, adapting only the layout source and the quantization ranges. The port reproduces the near-optimal performance its authors report (98% of FPLinQ) when trained and evaluated on their original stochastic channel model, confirming its fidelity.

The remaining learning-based schedulers surveyed in Section 2—e.g., the WMMSE-imitating DNN of Sun et al. [7], REGNN [8], the GNN framework of Shen et al. [9], UWMMSE [10], the DQN agents of Nasir and Guo [13], the decentralized GNN of Wang et al. [12], NAAC [14], and the centralized/distributed GCN schedulers of Zhao et al. [3]—do not appear in Table 3. The reasons are threefold: a mismatch in the decision variable, a mismatch in the channel model, and the scope of the evaluation reported here.

The first concerns what is being optimized, summarized in the “Decision Variable” column of Table 1. All of these methods except Zhao et al. optimize a continuous transmit power (or a beamformer, or a discrete power level), whereas the problem posed in (6) is the binary on/off scheduling problem. A power-allocation policy is not a scheduler: it may attain a higher sum-rate than any binary schedule simply by using a strictly larger action space, so placing it in Table 3 would compare two different problems rather than two solutions to one.

The second is channel-model mismatch. Every one of these methods except Zhao et al.’s was developed and tuned under a stochastic channel model—i.i.d. or block Rayleigh/Rician fading with a distance-dependent path-loss exponent—rather than the ray-traced Munich channels used here (Section 3.2); Zhao et al. operate instead on abstract conflict graphs with given per-link utilities, so evaluating their schedulers here would first require constructing a conflict graph and per-link utilities from H, a modeling step their authors neither specified nor validated. Each comes with training hyperparameters chosen for its own setting, and re-tuning them on a new channel distribution risks conflating algorithmic merit with reproduction quality in either direction.

The third is the scope of the present evaluation. Section 5.6 reports size-generalization results for both proposed schedulers across nine network sizes (N=2,…,10) from models trained only at N=10, since neither architecture requires per-size retraining. Matching this scope would mean retraining and validating a separate model at every size for baselines that are not size-invariant by construction (e.g., Sun et al.’s fixed-input DNN [7]); although the GNN-based baselines report permutation-equivariant transfer across sizes (REGNN [8], UWMMSE [10], Wang et al. [12]), that property was established under their own stochastic channel models and would itself need re-validation here. Porting any of these methods would therefore produce variants their authors never validated; their positioning relative to this work is instead established in Section 2 and Table 1. Accordingly, the comparisons reported in this paper support conclusions only about the binary scheduling formulation, the information assumptions, and the ray-traced setting considered here, not about the relative merit of learning-based methods designed for different formulations.

### 5.2. Evaluation Metrics

All methods are evaluated on the same 6000-snapshot test set at N=10 (Section 3.2.2). Unless stated otherwise, every scheduler is given the exact channel matrix H; this perfect-CSI assumption is relaxed in Section 5.7. We report (i) the average network sum-rate $R_{\mathrm{sum}}$ in bps/Hz; (ii) the percentage of the brute-force-optimal sum-rate achieved, $100\times {\bar{R}}_{\mathrm{sum}}/{\bar{R}}_{\mathrm{sum}}^{★}$; and (iii) the per-sample inference latency in microseconds, measured on an NVIDIA GeForce RTX 4090 GPU and, for comparison, on a single thread of a 13th-generation Intel Core i9 CPU (PyTorch 2.11), with explicit device synchronization around each timed batch of 128 snapshots. Each reported latency is the median over ten repetitions (five for brute force and FPLinQ) of the first 20 test batches (2560 snapshots); the interquartile ranges are given in Section 5.4. Latencies are intended for relative comparison across methods on a given platform rather than as absolute benchmarks. Accuracies of the learned schedulers are reported as the mean ± standard deviation over the five training seeds (Section 4.3), and the main differences between methods at N=10 are accompanied by seed-paired 95% confidence intervals (*t* distribution, four degrees of freedom), which reflect the variability across training runs on the fixed test set. To probe generalization to networks smaller than the training size, we additionally evaluate both proposed schedulers—trained only at N=10—on channel matrices sub-sampled (by uniformly random pair selection, without retraining) to N∈{2,3,…,9} from the same test snapshots, recomputing the brute-force-optimal, Greedy, Greedy + LS, and Highest-one references at each *N*. The same sub-sampled snapshots are also used to profile latency as a function of *N* (Section 5.4): for each *N*, latency is measured in the same way as at N=10.

### 5.3. Performance at the Training Size (N=10)

Table 3 reports sum-rate, percentage of optimal, and per-sample latency for all methods on the N=10 test set. Among the centralized methods, the proposed GNN alone reaches 94.60±0.13% of the brute-force-optimal sum-rate while requiring only 9.77 μs per sample on the GPU, 89× faster than exhaustive search (869 μs per sample), i.e., 1.1% of its computation time (on a single CPU thread, on which exhaustive search runs faster, the ratio is 25%, a 4× speed-up; Section 5.4); adding the local-search refinement raises this to 97.51±0.04% of optimal at a cumulative 103 μs per sample, still 8.4× faster than brute force. The proposed GNN also outperforms the Greedy heuristic both before local search (94.60% vs. 93.09%, a margin of 1.51 percentage points with a seed-paired 95% confidence interval of [1.36,1.67]) and after it (97.51% vs. 95.43% for Greedy + LS obtained under identical settings, a margin of 2.08 percentage points, 95% confidence interval [2.03,2.14]), indicating that the learned initial schedule provides a better starting point for local refinement than the hand-designed greedy rule. The seed-to-seed standard deviation of the proposed GNN, GNN + LS, and distributed schedulers, 0.04–0.27 percentage points, is an order of magnitude smaller than these margins. On the GPU, the GNN forward pass is also cheaper than the sequential greedy construction (9.77 vs. 34.4 μs), so GNN + LS is simultaneously more accurate and faster than Greedy + LS (103 vs. 121 μs), dominating the greedy pipeline on both axes on this platform; as Section 5.4 shows, this latency ordering does not hold on a single CPU thread.

The two literature baselines reinforce these comparisons. FPLinQ, given 1000 fixed fractional-programming iterations, reaches 90.54% of optimal—below even the greedy heuristic—because its iterations converge only to a stationary point of a continuous relaxation, which must ultimately be rounded to a binary schedule, and in a channel whose gains span five orders of magnitude this rounding is costly; the same local-search refinement lifts it to 95.27%, still 2.24 percentage points below GNN + LS at about twenty times the GPU latency (2070 vs. 103 μs). Its per-sample cost, dominated by the fixed iteration budget, in fact exceeds that of exhaustive search at this network size (2040 vs. 869 μs); since the fractional-programming iterations scale polynomially with *N* whereas exhaustive search scales as $2^{N}$, this ordering would reverse for larger networks, but at N=10 FPLinQ offers no latency advantage to offset its accuracy deficit. The graph-embedding scheduler of Lee et al. is, in contrast, the fastest centralized method in Table 3 (7.85 μs on the GPU) but reaches only 42.32±2.69% of optimal (five training seeds), far below the near-optimal performance it attains on its native stochastic channel model, where our port of the authors’ implementation reproduces their reported 98% of FPLinQ (Section 5.1). The collapse is most plausibly attributable to its features: quantized inter-node distances, chosen deliberately so that no CSI is required. In a ray-traced urban scene, building-induced shadowing decouples received power from distance, so the information those features carry no longer determines the schedule; neither more training data (58.06% of optimal with 16,000 training snapshots instead of 500, one training run) nor stronger supervision (brute-force-optimal labels in place of FPLinQ labels) recovers the loss, indicating that, in this setting, the limitation lies in the feature representation rather than in the training protocol. This observation is consistent with the channel-model gap that motivates this paper (a scheduler that is near-optimal under the stochastic, distance-driven channel model for which it was designed loses accuracy under site-specific propagation), but it is specific to the binary scheduling formulation, the distance-only input information, and the ray-traced scene considered here. It does not imply that the proposed schedulers are superior to learning-based methods that address different formulations or use different information, such as channel-based features, none of which were evaluated in this setting (Section 5.1).

Among the distributed methods, the proposed distributed scheduler reaches 70.70±0.27% of optimal using only pair-local information, exceeding the SIR-local heuristic (67.53%) by 3.17 percentage points (95% confidence interval [2.83,3.51]) and the Direct-threshold and All-on baselines (42.65% each; these two virtually coincide because essentially every pair’s stand-alone SNR exceeds 0 dB under the considered transmit power and link-distance constraint, so the naive SNR-threshold rule degenerates to “always transmit”) by 28.05 percentage points. Its GPU inference latency (10.6 μs/sample) is comparable to that of the centralized GNN, since both apply a similarly sized neural network, but its accuracy is substantially lower, reflecting the cost of deciding without access to the full interference pattern (Section 5.5 and Section 6).

**Figure 5:**
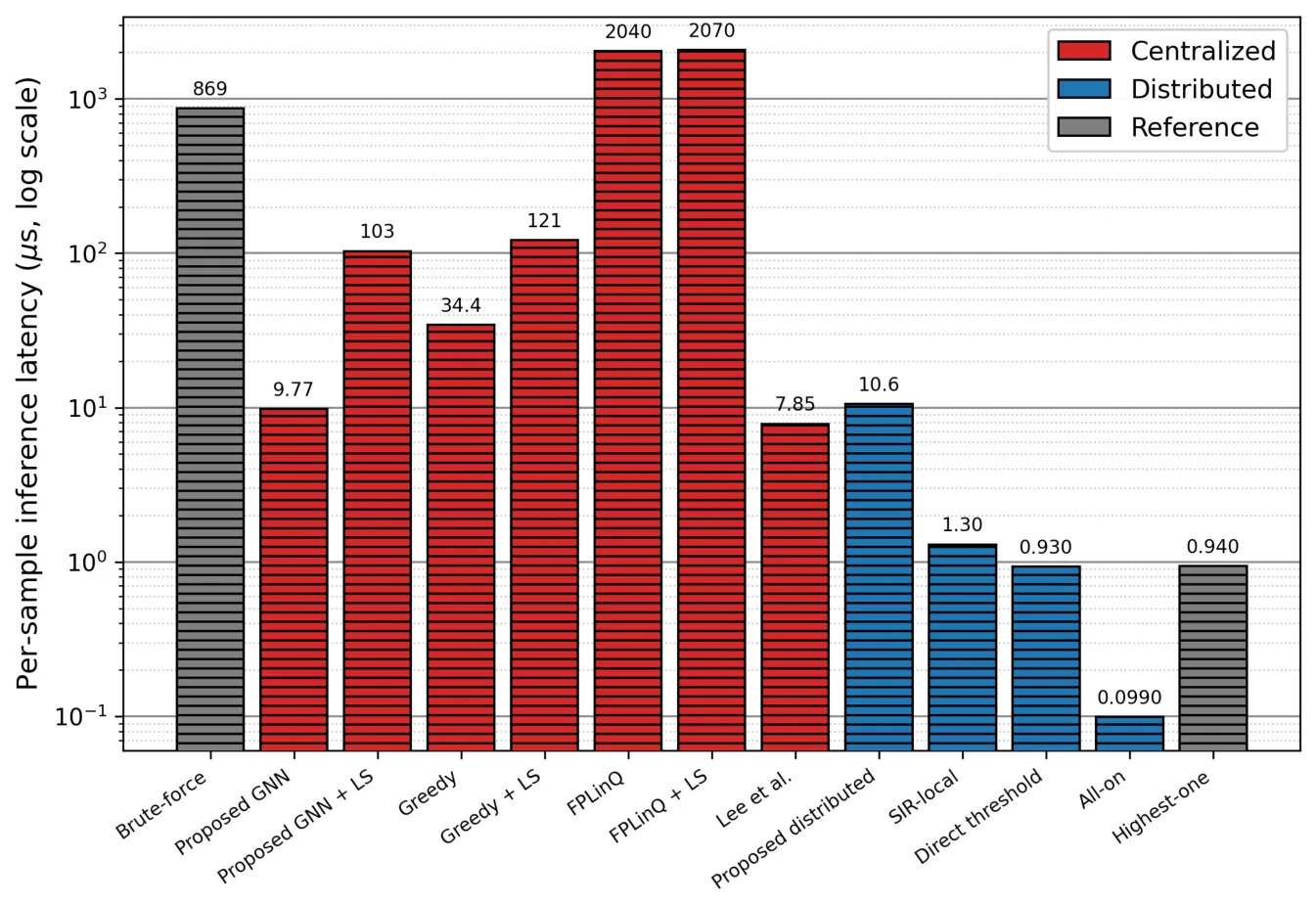
Per-sample inference latency (log scale) for all evaluated algorithms at N=10, corresponding to the latency column of Table 3 (RTX 4090 GPU, batch 128, median of ten repetitions, five for brute force and FPLinQ; $P_{\mathrm{tx}}^{\left(\mathrm{dBm}\right)}=23$ dBm).

Beyond sum-rate, per-sample inference latency is itself a practically important metric, since it determines how frequently a scheduler can be re-run as the network changes. Figure 5 visualizes the latency column of Table 3 on a logarithmic scale, showing that on the GPU brute-force search is slower than every other method by factors ranging from 8× (GNN + LS) to nearly 9000× (All-on), with the sole exception of the two FPLinQ variants, whose fixed 1000-iteration budget makes them the slowest methods in the table at this network size.

**Figure 6:**
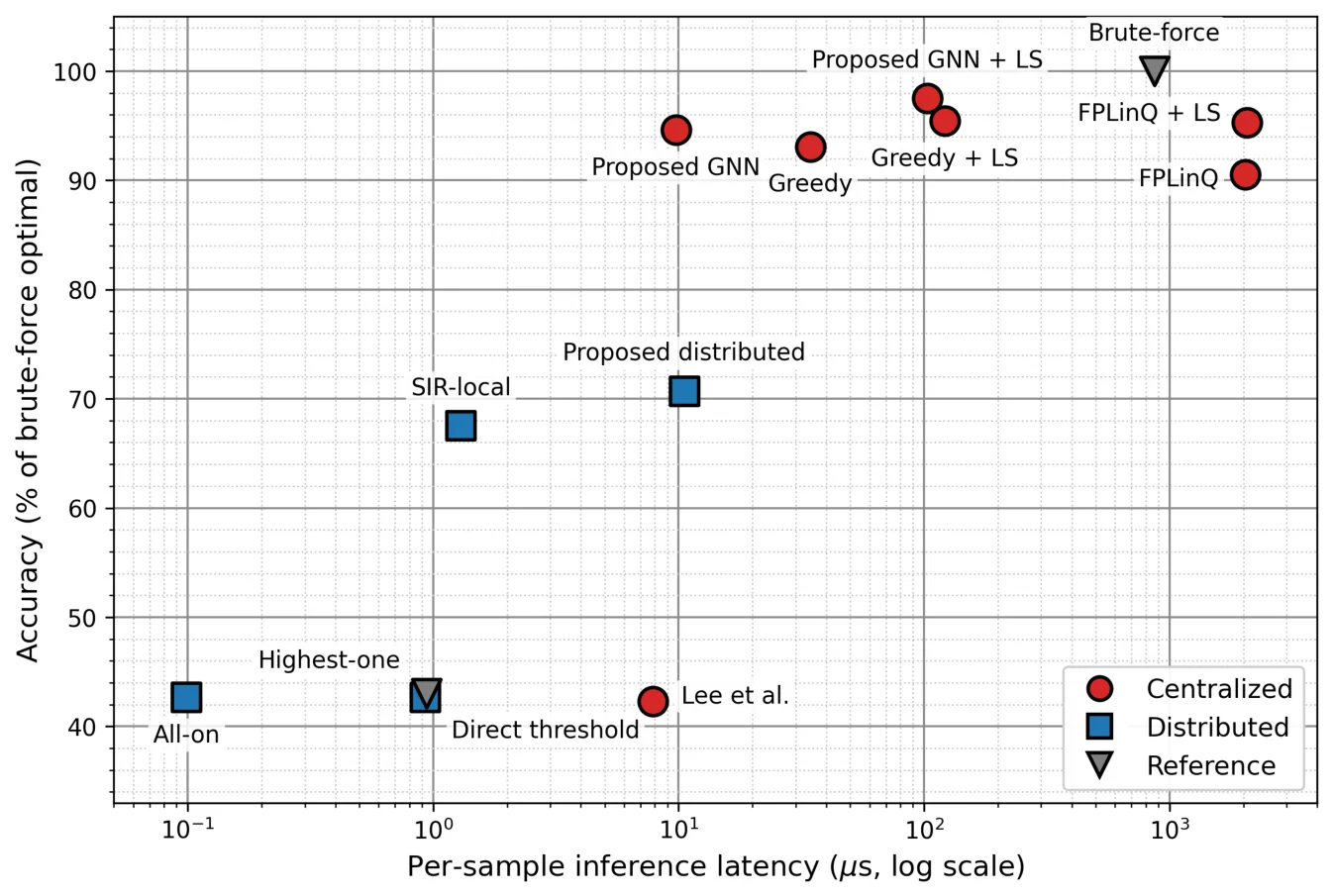
Accuracy–latency trade-off at N=10: sum-rate (% of brute-force optimal) versus per-sample inference latency (log scale) for all evaluated algorithms. Methods toward the upper-left corner offer the most favorable trade-off. Learned schedulers: mean over five training seeds; latency on the RTX 4090 GPU.

Figure 6 re-plots the same data as sum-rate (% of optimal) against GPU latency, which makes the accuracy–latency trade-off explicit: brute-force sits in the top-right corner (optimal but slow), the coordination-free distributed heuristics (All-on, Direct threshold, Highest-one) sit in the bottom-left (fast but far from optimal), and the proposed schedulers occupy the favorable upper-left region, combining low latency with high accuracy; the FPLinQ variants fall in the slow right half alongside brute force, while the Lee et al. scheduler joins the fast-but-inaccurate group in the lower left despite being centralized. Within the centralized family, GNN + LS Pareto-dominates Greedy + LS on this platform (97.51% at 103 μs versus 95.43% at 121 μs, i.e., strictly higher accuracy at strictly lower latency) and dominates FPLinQ + LS by a still wider margin on both axes; within the distributed family, the proposed scheduler is more accurate than SIR-local and Direct threshold at a latency of the same order as that of the centralized GNN.

### 5.4. Computational Complexity: Latency Scaling with Network Size

**Figure 7:**
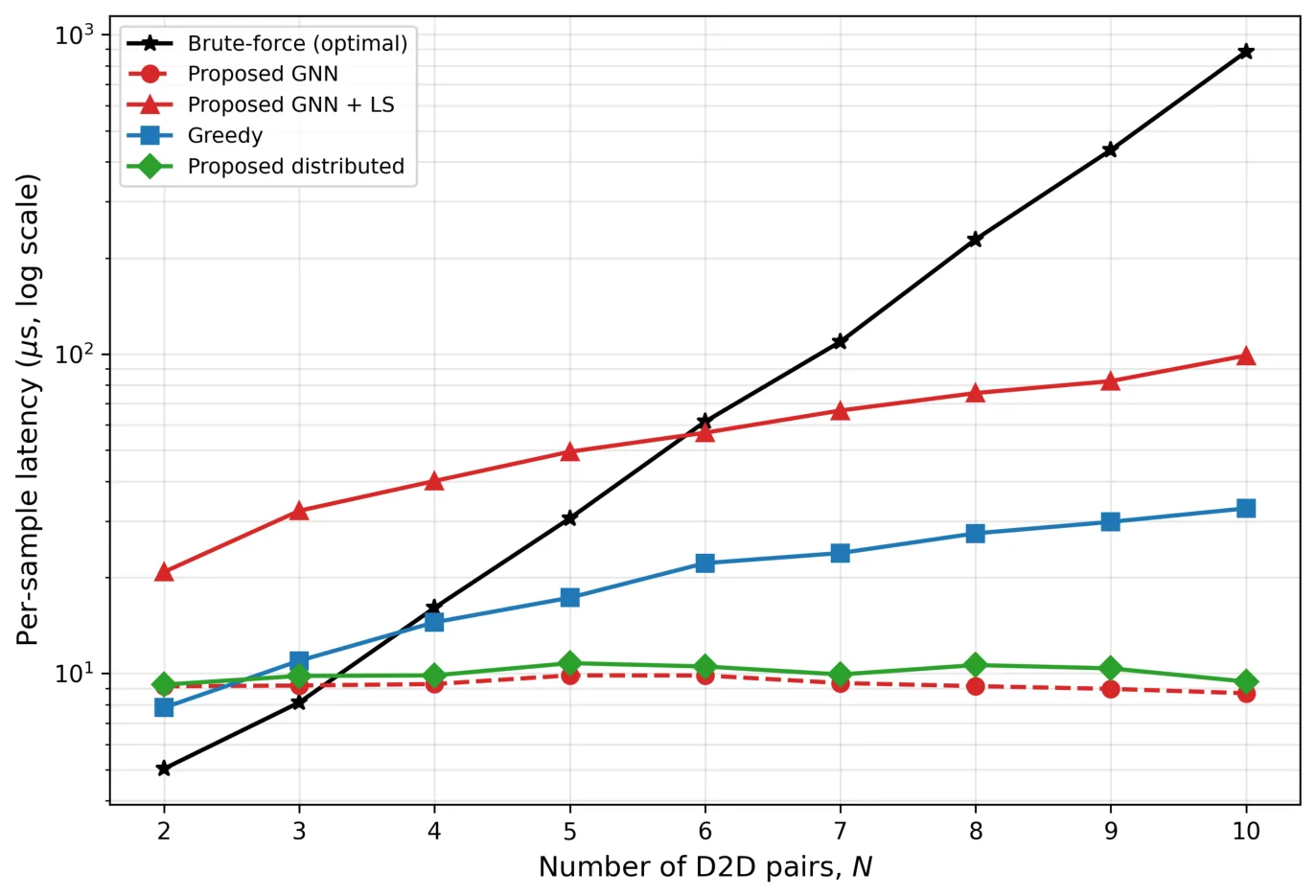
Per-sample inference latency versus number of D2D pairs *N* (log scale), evaluated on the same sub-sampled snapshots as Figure 8 (model trained at N=10). Brute-force latency grows exponentially with *N*, while the proposed centralized GNN forward pass and distributed scheduler remain nearly flat; the GNN + LS curve rises with *N* due to the growing cost of local-search refinement rather than the network itself. RTX 4090 GPU, batch 128, median of ten repetitions (five for brute force).

The N=10 snapshot in Table 3 is only one point on a much steeper trend: because brute-force search evaluates $2^{N}$ candidate schedules, its latency grows exponentially with *N*, whereas every learned or greedy alternative grows at most polynomially. Extending the timing measurement to N=2,…,10 using the same sub-sampled snapshots described in Section 5.2 makes this divergence explicit (Figure 7). On the GPU, brute-force latency roughly doubles with every additional pair, from 5.0 μs at N=2 to 884 μs at N=10, while the proposed centralized GNN (forward pass only) and distributed scheduler both remain essentially flat across the entire range (8.7–10.8 μs), although the arithmetic cost of the centralized scheduler grows as $O(N^{2})$ (Section 4.1): at these small sizes, the GPU executes the forward pass in parallel and its latency is dominated by fixed per-call overheads rather than by arithmetic; Greedy increases roughly fourfold over the same range (7.8 μs at N=2 to 32.9 μs at N=10), reflecting its O(N) sequential activation steps. Brute-force is faster than the proposed GNN only for the smallest networks tested (N≤3); from N=4 onward, the GNN is already faster than exhaustive search, and the gap widens rapidly with *N*. Unlike the GNN forward pass itself, the local-search refinement’s added cost is not *N*-independent: the cumulative latency of GNN + LS rises from 20.8 μs at N=2 to 98.9 μs at N=10, since each of its up to *N* sweeps re-evaluates all *N* candidate bit flips, making the refinement step—rather than the underlying network—the main driver of the mild growth visible in Figure 7. Within the tested range (N≤10), the favorable accuracy–latency trade-off observed at N=10 is therefore not specific to that size. Beyond this range, the exponential growth of exhaustive search will continue to dominate, but the quadratic cost of the centralized scheduler will eventually outgrow the fixed GPU overheads, so its latency advantage at larger *N* cannot be inferred from these measurements. The absolute latencies of this *N*-sweep differ slightly from the corresponding entries of Table 3 (e.g., 884 vs. 869 μs for brute-force at N=10) because the two are separate timing runs of the same quantity; only the sum-rate and percentage-of-optimal figures are deterministic and therefore identical across runs. Latencies should accordingly be compared across methods within a run, not across runs.

The latency ordering reported so far is specific to batched execution on a GPU. Table 4 repeats the measurement on a single CPU thread and adds the latency of scheduling a single network (batch size 1), including the cost of one distributed decision, i.e., of one pair evaluating its MLP on its own feature vector, which is what a device executes at run time. On the CPU, the centralized GNN (109 μs per sample) is 13 times slower than the greedy heuristic (8.26 μs) and only four times faster than exhaustive search (433 μs), which itself runs faster on the CPU than on the GPU because our implementation evaluates the $2^{N}=1024$ candidate schedules one after another, as small operations whose GPU launch overhead exceeds their arithmetic, and GNN + LS no longer dominates Greedy + LS in latency (134 vs. 32.4 μs): the accuracy advantage of the learned schedulers does not depend on the platform, but their latency advantage over lightweight heuristics relies on parallel hardware. For a single network, per-call overheads dominate on the GPU (about 1.2–1.5 ms for either scheduler, regardless of *N*), whereas on the CPU, the latency of the centralized scheduler grows with *N*, from 0.45 ms at N=10 to 9.4 ms at N=100, as its $O(N^{2})$ arithmetic increasingly outweighs the fixed per-call cost. By contrast, one distributed decision takes about 40 μs on a single CPU thread, independently of *N*; this time is dominated by the per-call overhead of the software framework rather than by the 0.10 MFLOPs of arithmetic per pair (Section 4.2). This value was measured on a desktop CPU core and is only indicative of the latency on a battery-powered device, whose processor is slower, but it shows that the per-device cost is small and does not grow with the size of the network. Over the ten repetitions, the interquartile range of the GPU latencies in Table 3 is within 10% of the median for all methods except the distributed scheduler and the Direct-threshold rule (28% and 14%, respectively), whose GPU timings showed larger run-to-run jitter; on the CPU, it is below 8% for all methods in Table 3.

### 5.5. Centralized–Distributed Trade-Off

The performance gap between the centralized and distributed proposed schedulers is 23.90 percentage points (94.60% vs. 70.70%; seed-paired 95% confidence interval [23.59,24.22]), widening to 26.81 percentage points if the centralized model’s local-search upper bound (97.51%) is used as the reference. For context, applying the same 1-bit-flip local search to the distributed scheduler’s output raises it to 92.50±0.46% of optimal, but this post-processing step requires centralized recomputation of the exact sum-rate at every candidate flip and therefore violates the distributed information constraint; we report it only as a diagnostic upper bound on how much residual sum-rate is recoverable from the distributed scheduler’s initial decisions, and exclude it from Table 3. The distributed model is also the more compact of the two (52,226 vs. 64,836 parameters), so the accuracy gap is unlikely to be explained by the modest difference in model size; a more plausible contributor is the information lost by deciding from row/column statistics only rather than the complete interference graph. The gap is itself a function of network size, widening from 8.57 percentage points at N=2 to 23.90 at N=10 (Section 5.6): the value of full coordination grows as more pairs compete for the shared spectrum.

To test whether this gap stems from the particular choice of local features or from the information that local features can carry at all, we retrained the distributed scheduler with subsets of its 19-dimensional input (Table 5; three seeds each, all other settings unchanged): the direct-link gain alone; the direct gain with the nine row statistics (received-interference side) or with the nine column statistics (transmitted-interference side); the row and column statistics without the normalized direct gain; and the full set. At N=10, the direct gain alone attains 62.05% of optimal. Adding either the row or the column statistics raises accuracy to about 69.5%, and the two are nearly interchangeable; combining them adds only a further 1.4 percentage points, to 70.87% for the full set (70.49% without the direct-gain feature, whose information is largely contained in the SIR-type row and column statistics). Within the family of row and column statistics considered here, local information therefore saturates at about 71% of optimal, 23.7 percentage points below the centralized scheduler: enriching what a pair measures about its own links yields rapidly diminishing returns, whereas what it cannot observe, namely the decisions of the other pairs, is precisely what the centralized scheduler exploits. These results are consistent with information asymmetry as a major contributor to the centralized–distributed gap, but they do not establish it as the only cause: the ablation varies the inputs only within this family of statistics, so local information of another kind or a different architecture of the per-pair policy might still narrow the gap (Section 6).

### 5.6. Sum-Rate Generalization to Smaller Networks (N≤10)

**Figure 8:**
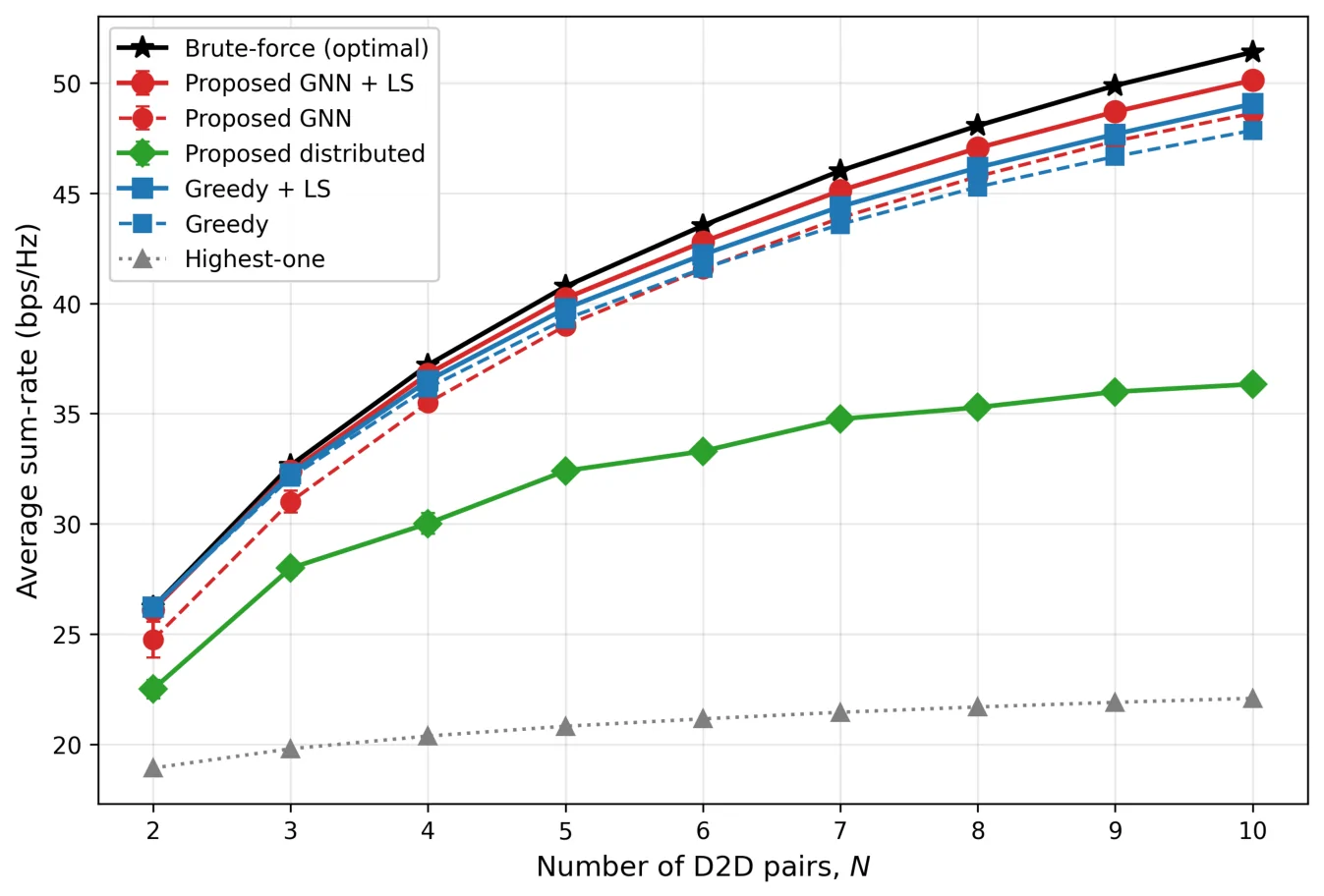
Average sum-rate versus number of D2D pairs *N* for the centralized GNN scheduler, the distributed scheduler (both trained only at N=10), and baseline methods, evaluated on sub-sampled test snapshots. Learned schedulers: mean over five training seeds, error bars ±1 standard deviation; $P_{\mathrm{tx}}^{\left(\mathrm{dBm}\right)}=23$ dBm.

**Figure 9:**
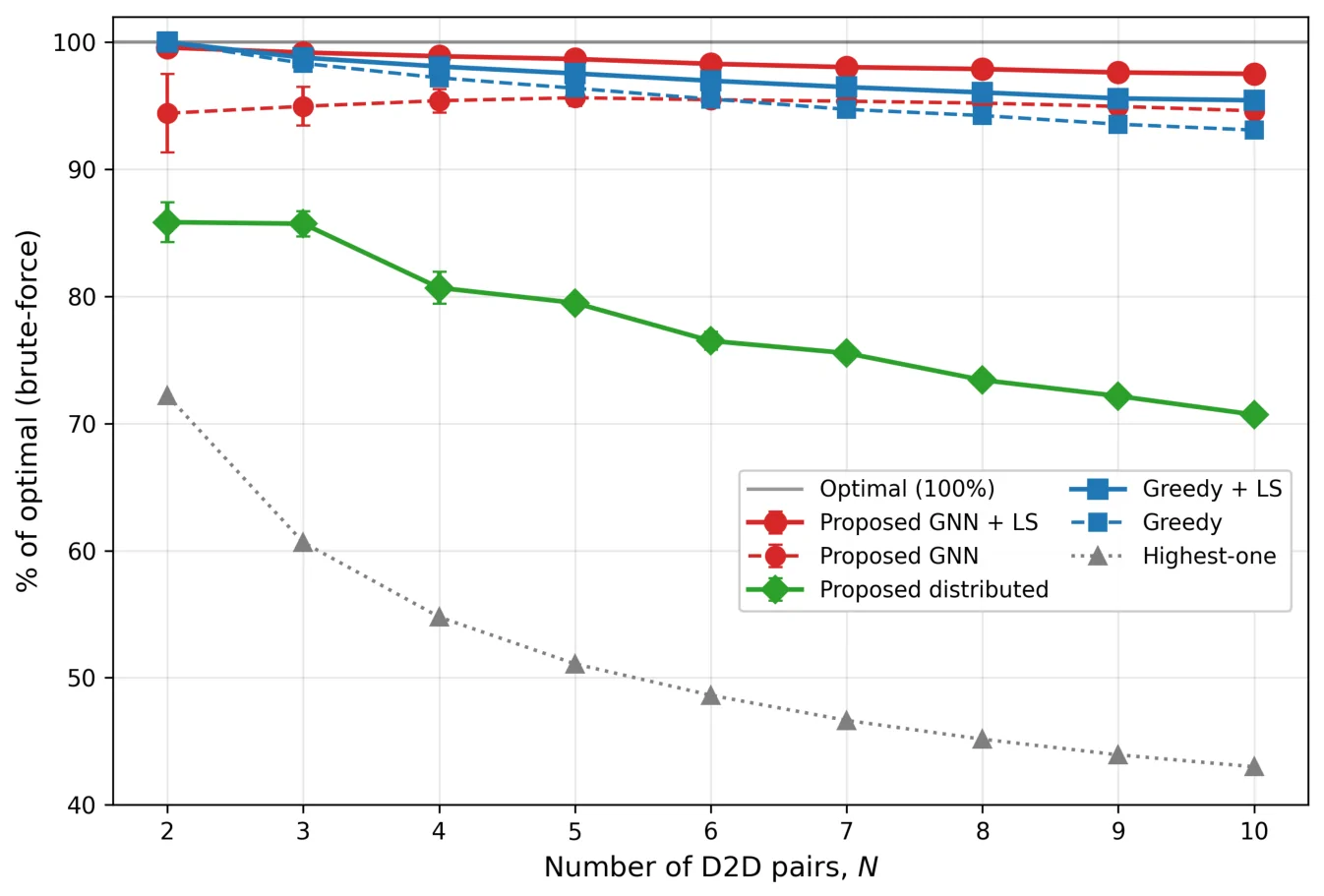
Percentage of the brute-force-optimal sum-rate versus number of D2D pairs *N*, corresponding to Figure 8 (mean ± standard deviation over five training seeds).

While Table 3 fixes N=10, Table 6 and Figure 8 and Figure 9 report how sum-rate accuracy changes when the same centralized GNN—trained only at N=10—is evaluated, without retraining, on sub-sampled networks with N=2,…,9 pairs. The GNN alone stays within a narrow 94.4–95.6% band of the (re-computed, exact) brute-force optimum across the entire range, a total variation of less than 1.3 percentage points. Its accuracy is not monotone in *N*: it rises from 94.41% at N=2 to a maximum of 95.63% at N=5 and then declines gently to 94.60% at N=10, so its worst case over the sweep occurs at the smallest network rather than the largest. The dip at very small *N* is expected, since with only two or three pairs there is almost no interference structure for the attention mechanism to exploit; the smallest networks are also where the result depends most on the training run: the seed-to-seed standard deviation of the GNN grows from 0.13 percentage points at N=10 to 3.09 at N=2 (Table 6), whereas that of GNN + LS stays below 0.3 percentage points at every size. What matters for the generalization claim is that the policy neither degrades with distance from its training size nor exhibits any systematic drift, but stays flat within 2 percentage points across a five-fold change in network size. These results establish generalization only toward networks smaller than the training size; whether either scheduler extrapolates to N>10 is not evaluated in this paper (Section 6). The GNN + LS combination stays above 97.5% of optimal at every tested size and is the best-performing method at every N≥3, ahead of Greedy + LS by 0.4–2.1 percentage points, with the margin widening as *N* grows; only Greedy and Greedy + LS, both at 100.00% for N=2, surpass it, and then by just 0.42 percentage points. Both figures show the expected trend that the naive Highest-one baseline degrades sharply as *N* grows (from 72.20% at N=2 to 42.99% at N=10), since ignoring all but one pair becomes increasingly wasteful as more pairs could be scheduled concurrently.

The distributed scheduler—also trained only at N=10—likewise requires no structural modification to run at other sizes, since its local feature vector has a fixed dimensionality regardless of *N*, and Table 6 and Figure 8 and Figure 9 include it (without local-search post-processing, consistent with Table 3) alongside the centralized methods. It outperforms the naive Highest-one baseline at every tested size, but its accuracy trend is qualitatively different from the centralized GNN’s: rather than staying flat, it declines monotonically from 85.84% of optimal at N=2 to 70.70% at N=10. The centralized–distributed accuracy gap therefore widens from 8.57 percentage points at N=2 to 23.90 percentage points at N=10, a trend discussed further in Section 6.

### 5.7. Robustness to Imperfect Channel State Information

#### 5.7.1. Independent Estimation Error

All results reported so far assume that the scheduler is given the exact channel matrix H. In practice, H is estimated from pilot transmissions and is therefore corrupted by estimation error—a particularly relevant concern for the centralized scheduler, whose decisions depend on all $N^{2}$ entries of H rather than on the 2N−1 entries a single pair can measure for itself.

We adopt a power-preserving error model, which has the same form as the first-order Gauss–Markov model widely used for channel estimation error and channel aging [34], applied entry-wise so that every link is subject to the same relative error:

(12) $$\hat{H}\left[i,j\right]\;=\;\sqrt{1-\varepsilon^{2}}\;H\left[i,j\right]\;+\;\varepsilon \;Z\left[i,j\right],\;Z\left[i,j\right]∼\mathrm{CN}\;\left({0,\;|H\left[i,j\right]|}^{2}\right),$$

where $\varepsilon^{2}\in \left[0,1\right]$ is the per-link normalized mean-square estimation error, so that $\varepsilon^{2}=0$ recovers perfect CSI and $\varepsilon^{2}=0.5$ corresponds to an estimate correlated with the true coefficient by $\sqrt{1-\varepsilon^{2}}=0.707$. Because the perturbation variance is scaled by ${\left|H\left[i,j\right]\right|}^{2}$ rather than by a matrix-wide constant, the model preserves the received power on every link individually, $E{\left|\hat{H}\left[i,j\right]\right|}^{2}={\left|H\left[i,j\right]\right|}^{2}$, and does not disproportionately corrupt the weak entries. This matters in a ray-traced dataset: the entries of ${\left|H\right|}^{2}$ span roughly five orders of magnitude between their mean and their median, so a batch-wide constant noise variance would replace the majority of entries with pure noise and confound the CSI-robustness question with an artifact of the scaling convention. Entries for which the ray tracer resolves no propagation path, H[i,j]=0 (38% of all entries, and 42% of the off-diagonal cross-link entries; direct links always have at least one resolved path by construction, Section 3.2.2), are left at zero, since a link that does not exist cannot be mis-estimated.

The perturbed matrix $\hat{H}$ is used only as the scheduler’s input: the sum-rate achieved by the resulting schedule is always evaluated on the true channel H via (5), and the brute-force optimum in the denominator is likewise computed from H and therefore remains fixed at 51.3911 bps/Hz for every $\varepsilon^{2}$. All methods that consume channel information receive the same realization of $\hat{H}$ at each $\varepsilon^{2}$, so the comparison is matched; the distributed scheduler’s local features are extracted from the corresponding row and column of $\hat{H}$. The two literature baselines of Table 3 are omitted from this sweep: the Lee et al. scheduler consumes only inter-node distances and is therefore unaffected by channel estimation error by construction, while FPLinQ’s perfect-CSI accuracy already places it below the methods whose robustness ordering is at issue.

**Figure 10:**
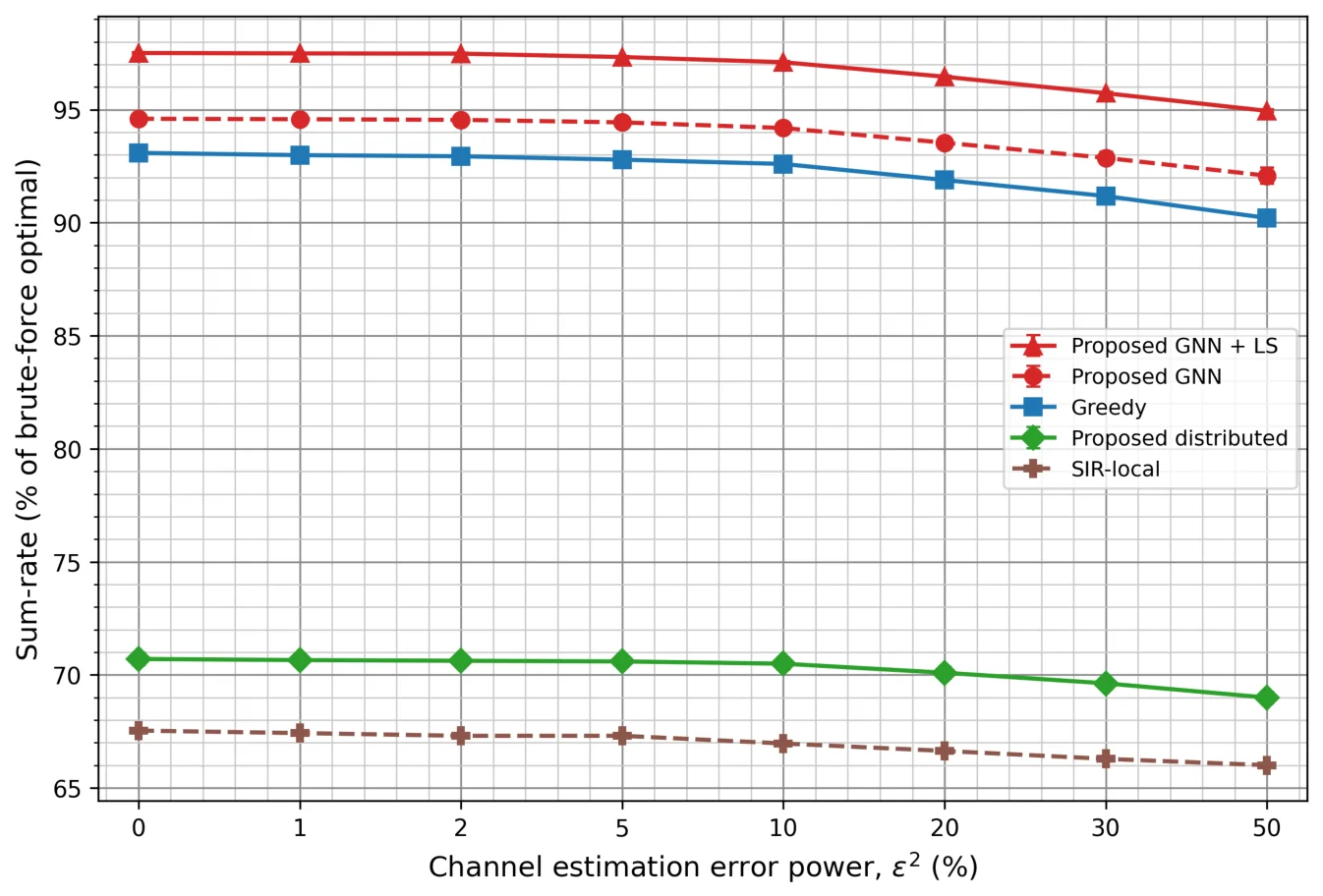
Percentage of the brute-force-optimal sum-rate versus the per-link normalized channel estimation error $\varepsilon^{2}$ at N=10, corresponding to Table 7. The horizontal axis is labeled in percent, so its tick at 1 corresponds to $\varepsilon^{2}=0.01$ in Table 7; the sampled values are drawn at equal spacing, so the axis is not linear in $\varepsilon^{2}$. The All-on and Direct-threshold baselines are omitted: All-on ignores the channel entirely, and every pair’s stand-alone SNR exceeds the 0 dB threshold by so wide a margin that the perturbations considered here flip virtually no decisions, so both remain at 42.65% throughout and would only extend the vertical range. Learned schedulers: mean over five training seeds, error bars ±1 standard deviation.

Table 7 and Figure 10 report the resulting percentage of optimal over $\varepsilon^{2}\in \left\{0,0.01,0.02,0.05,0.10,0.20,0.30,0.50\right\}$ at N=10 on the same 6000-snapshot test set. Two observations stand out. First, the degradation is graceful throughout: even at $\varepsilon^{2}=0.5$, every method in Table 7 retains 96.9–97.7% of its own perfect-CSI accuracy, and the proposed centralized GNN loses only 2.52 percentage points (94.60% to 92.08%) over the entire sweep. Second, the ranking of the methods is preserved at every error level, so none of the conclusions drawn in Section 5.3, Section 5.4, Section 5.5 and Section 5.6 is contingent on the perfect-CSI assumption under this error model; Section 5.7.2 examines impairments that it does not capture.

The robustness to independent errors can be attributed to the discrete nature of the scheduling decision. The schedule depends on the *ordering* of link gains rather than on their precise values, and in a ray-traced urban environment that ordering is separated by orders of magnitude: a multiplicative perturbation must be very large before it reverses the relative standing of two links that differ by 30 dB. We therefore expect a scheduler to be intrinsically less sensitive to relative channel error than a rate- or power-allocation scheme that consumes the gain values directly. This argument applies to errors on channel entries that are present; it does not extend to entries that are missing altogether, which affect both learned schedulers far more strongly (Section 5.7.2).

It should be noted that the distributed scheduler’s smaller absolute drop (1.71 percentage points, 70.70% to 68.99%) does *not* indicate greater robustness: measured relative to its own perfect-CSI accuracy, its retention (97.6%) is essentially indistinguishable from the centralized GNN’s (97.3%), and the smaller absolute loss simply reflects its lower starting point. Independent estimation error therefore degrades centralized and distributed operation by essentially the same *fraction*, leaving the trade-off analyzed in Section 5.5 unchanged rather than tilted.

#### 5.7.2. Silent Peers, SNR-Dependent Error, Quantization, and Channel Aging

The error model (12) perturbs every entry independently and by the same relative amount. We now relax it in the directions that the acquisition procedure of Section 4.2 makes relevant (Table 8), again feeding every scheduler the same corrupted input and evaluating the achieved sum-rate on the true channel; results are expressed as the percentage of each method’s own perfect-CSI accuracy that it retains.

*Silent peers.* Because the rows and columns of H are acquired by overhearing, a pair that has not transmitted during the estimation window leaves the cross-link entries of its own row and column unobserved, both by the other pairs and by a controller. We let a fraction *q* of the pairs, drawn independently for every snapshot, be silent, and either set their unobserved cross-link entries to zero (i.e., treat an unheard interferer as absent) or replace them by stale estimates, modeled by (12) with $\varepsilon^{2}=0.5$ (a correlation of 0.71 with the current channel). Treating unobserved interferers as absent is harmful for every scheduler that uses cross-link information, and most harmful for the centralized ones: with 30% silent pairs, the GNN retains only 61.1% of its perfect-CSI accuracy, against 76.4% for the distributed scheduler, whose decision depends only on the pair’s own row and column and is therefore affected only when its own interferers are silent. In absolute terms, however, the centralized scheduler remains the more accurate of the two (57.8% versus 54.0% of optimal at q=0.3), and at q=0.3, neither learned scheduler retains its advantage over the corresponding heuristic (Greedy 58.6%, SIR-local 54.5%). Using stale estimates instead removes almost the entire loss: even with half of the pairs silent, every method retains at least 98.5% of its accuracy. A pair or a controller should therefore keep its most recent estimate of an unheard link rather than discard it, at least as long as that estimate is no older than the staleness evaluated here ($\varepsilon^{2}=0.5$, about 20 ms at pedestrian speed; see below).

*SNR-dependent estimation error.* In practice, the estimation quality of a link degrades with its received power. Setting the per-entry error in (12) to $\varepsilon_{ij}^{2}={\left(1+G_{p}P_{\mathrm{tx}}{\left|H\left[i,j\right]\right|}^{2}/P_{n}\right)}^{-1}$, the error of a pilot with processing gain $G_{p}$, leaves every scheduler within 0.1% of its perfect-CSI accuracy for $G_{p}\geq 0$ dB; even an unrealistically poor pilot ($G_{p}=-30$ dB, an additional stress test) costs at most 1.2%. The weak cross-links that such errors corrupt most are precisely those that barely influence the schedule.

*Quantization.* When the gains are reported through a uniform quantizer over [−140,−40] dB, 4-bit reporting retains at least 98.9% of every method’s accuracy and 3-bit reporting at least 96.1%; only 2-bit reporting costs 5–9%.

*Channel aging.* Because (12) has the form of a first-order Gauss–Markov model [34], the $\varepsilon^{2}$ sweep of Table 7 can also be read as a sweep over the age of the estimate: with the Jakes correlation $\rho =J_{0}\left(2\pi f_{D}\tau \right)$ and $\varepsilon^{2}=1-\rho^{2}$, a pedestrian at 3 km/h ($f_{D}=8.3$ Hz at $f_{c}=3$ GHz) reaches $\varepsilon^{2}=0.13$ after τ=10 ms and 0.45 after 20 ms, whereas at 30 km/h $\varepsilon^{2}=0.13$ is already reached after 1 ms. The evaluated range $\varepsilon^{2}\leq 0.5$ thus corresponds to estimates up to about 20 ms old when one device of a link moves at pedestrian speed, or about 15 ms when both do (correlation $J_{0}^{2}\left(2\pi f_{D}\tau \right)$); faster movement or longer feedback delays fall outside it. Because the snapshots of our dataset are temporally independent, channel aging along actual device trajectories could not be simulated (Section 6).

### 5.8. Sensitivity to the Operating Point

All results so far are obtained at the conducted transmit power of 23 dBm (Table 2). Because the schedulers observe only the normalized channel gains, a trained model produces the same schedule at any transmit power, whereas the optimal schedule changes: at low SNR, noise dominates and more pairs can be activated, while at high SNR, interference dominates. Table 9 evaluates the models trained at 23 dBm, without retraining, for transmit powers from 17 to 29 dBm. Across this range, the GNN stays within 93.9–94.6% of optimal, GNN + LS within 97.0–97.8%, and the distributed scheduler within 68.1–72.9%. Their advantage over the corresponding heuristics grows with the transmit power, i.e., with the importance of interference. At 17 dBm, 6 dB below the training point, Greedy (94.43%) is on par with the GNN (94.35%, within its seed-to-seed standard deviation of 0.18 percentage points), although GNN + LS still exceeds Greedy + LS (97.76% vs. 96.47%). A deployment far from the training operating point would therefore benefit from retraining at, or across, the relevant range of transmit powers.

## 6. Discussion

The results in Section 5 support four main observations.

First, an attention-based GNN trained purely with a sum-rate reward, without any labeled optimal schedule, reaches within a few percentage points of the brute-force optimum while running roughly 90× faster on a GPU and 4× faster on a single CPU thread, and a cheap local-search refinement closes most of the remaining gap at a cost that is, on a GPU, still below that of the greedy heuristic followed by the same refinement; on a single CPU thread, the greedy pipeline is faster (Section 5.4). This makes the centralized design a practical option whenever a controller with access to the full channel matrix is available.

Second, the size-generalization results (Table 6, Figure 8 and Figure 9) show that this benefit is not confined to the training size as long as the network does not exceed it: a single model trained at N=10 stays within a 94.4–95.6% band of optimal across N=2 to N=10 without retraining, which is attractive for D2D deployments where the active pair count fluctuates over time below a known maximum.

Third, the comparison between the centralized and distributed schedulers (Section 5.5) quantifies a trade-off that is often only discussed qualitatively in the literature: restricting a scheduler to information locally available to each pair—removing the signaling overhead of collecting the full channel matrix at a controller—costs roughly 24 percentage points of sum-rate optimality in our setting, even though the distributed model still outperforms the coordination-free heuristics by a wide margin and the stronger SIR-local heuristic by a smaller but consistent one. The feature ablation of Section 5.5 suggests that information asymmetry is a major contributor to this loss: a pair cannot know, from its own row and column of H alone, whether another pair with which it does not directly interact will also transmit, so coordinated but non-adjacent activation patterns available to the centralized scheduler are unavailable to it. Consistent with this, adding row statistics, column statistics, or both to the pair’s own direct gain yields rapidly diminishing returns, and the accuracy of the distributed scheduler saturates at about 71% of optimal. These observations do not exclude other contributors, such as the architecture of the per-pair policy or local information outside the family of statistics considered here. That applying centralized local search to the distributed scheduler’s initial decisions recovers most of this gap (92.5% of optimal) shows that most of this gap is recoverable by coordination; since local search is itself a centralized optimizer, however, this bounds the recoverable sum-rate rather than identifying the cause of the gap.

Fourth, the imperfect-CSI study of Section 5.7 qualifies the cost of the centralized scheduler’s reliance on the full channel matrix. Under independent estimation error, SNR-dependent estimation error, stale estimates, and quantized reporting down to 3 bits, the centralized and distributed schedulers lose a similar and small fraction of their accuracy, so, under these impairments, an imprecisely acquired channel matrix does not penalize the centralized scheduler more than the distributed one. Missing entries are different: if the entries of silent peers are treated as zero, the centralized scheduler loses a markedly larger fraction of its accuracy than the distributed one (retaining 61% versus 76% of its perfect-CSI accuracy with 30% silent pairs). In absolute terms, the centralized scheduler nevertheless remains the more accurate one, but once 30% or more of the pairs are silent, neither learned scheduler retains its advantage over the corresponding heuristic. Incompletely acquired CSI therefore erodes the benefit of learning more than it changes the centralized–distributed ranking, and both a controller and a pair should retain the most recent estimate of every link, as long as it is not too old, rather than discard links they have not recently observed.

This study has several limitations that suggest directions for future work.

First, the channel model, while ray-traced and geometry-consistent, is restricted to a single scene (downtown Munich), a single carrier frequency (3 GHz), and static, temporally uncorrelated snapshots; extending the dataset to multiple scenes, frequency bands, and temporally correlated trajectories would test the robustness of the learned policies more broadly.

Second, the scheduling decision is restricted to a binary on/off model at fixed transmit power; combining the proposed schedulers with continuous power control, as commonly studied for centralized GNN policies [9], is a natural extension.

Third, the perfect-CSI assumption that underlies Section 5.3, Section 5.4, Section 5.5 and Section 5.6 has been relaxed only partially. Section 5.7 covers independent and SNR-dependent estimation error, silent peers with missing or stale estimates, and quantized reporting, and it relates the independent-error sweep to channel aging through the Gauss–Markov model; it does not capture estimation errors that are correlated across links, nor channel aging along actual device trajectories, because the snapshots of our dataset are temporally independent. Nor does it account for the signaling overhead of collecting H at a controller, which remains the principal practical argument for distributed operation.

Fourth, the distributed scheduler’s accuracy, unlike the centralized model’s, does not hold up across network sizes (Section 5.6). Because its local feature vector is computed from row/column statistics that are already size-invariant in form, it might have been expected to generalize across *N* in the same way as the centralized model; the empirical result is less favorable. It does run without retraining at every tested size and consistently outperforms the coordination-free baselines, but its percentage of optimal declines monotonically with *N* (85.84% at N=2 to 70.70% at N=10), so the centralized–distributed gap is not a fixed cost but one that grows with network size.

Fifth, stronger training-time supervision does not narrow the gap. Both distributed models are trained centrally and executed by each pair independently, i.e., both follow the centralized-training/decentralized-execution (CTDE) paradigm (Section 4.2 and Section 4.3), which rests on the premise that information available only at training time can usefully shape a policy that must later execute independently [21]; the two models differ only in their training signal. The REINFORCE-only distributed model learns from the sum-rate reward alone, whereas the imitation-pretrained distributed model is first pre-trained to imitate brute-force-optimal labels, which are available only at training time, and then fine-tuned with REINFORCE (Section 4.3). The imitation-pretrained model reaches 70.37±0.49% of optimal at N=10 over five training seeds, 0.33 percentage points below the REINFORCE-only model; this difference is not statistically distinguishable from zero (seed-paired 95% confidence interval [−0.82,+0.16]), and both variants share the same architecture and hence the same inference cost. Together with the diagnostic local-search result and the feature ablation of Section 5.5, this parity suggests that the gap is not caused mainly by the difficulty of optimizing the policy from a sum-rate reward alone: a stronger supervised training signal changes how the policy is trained but not what it can see. It does not, however, rule out that a different architecture of the per-pair policy or local information of another kind could narrow the gap.

Sixth, the learning-based comparison covers the one prior method that shares our binary decision variable and operates on the physical geometry of the network rather than on an abstract conflict graph (Lee et al. [11]); porting the remaining schedulers of Section 2 to the binary, ray-traced setting—as well as re-training the Lee et al. scheduler with channel-based rather than distance-based features, which its authors deliberately avoided and whose benefit in this environment remains to be established—remains future work.

Seventh, both schedulers were trained at N=10 and evaluated only for N≤10. Their behavior in networks larger than the training size, where the $O(N^{2})$ attention cost of the centralized scheduler (Section 4.1) also becomes a practical concern, remains to be evaluated.

Finally, latencies were measured on one desktop GPU and on one desktop CPU core (Section 5.4) and are intended for relative comparison; the latency ordering between the learned schedulers and lightweight heuristics differs between the two platforms, and the latency of the distributed scheduler on an actual mobile or IoT device, whose processor is slower than a desktop CPU core, remains to be measured.

## 7. Conclusions

This paper studied graph neural network-based scheduling for interference-limited D2D networks, trained and evaluated on channel data obtained from Sionna ray tracing over a real three-dimensional city scene rather than from a stochastic fading model. A centralized graph-attention scheduler, trained end-to-end with a sum-rate policy-gradient reward and no labeled optimal schedule, reached 94.6% of the brute-force-optimal sum-rate (97.5% with a lightweight local-search refinement) using only 1.1% of the computation time of exhaustive search on a GPU (25% on a single CPU thread), and it held a narrow 94.4–95.6% band of optimality across network sizes from 2 to 10 pairs despite being trained only at 10 pairs, i.e., for networks no larger than the training size. A fully distributed counterpart, restricted to information each D2D pair can measure locally with no inter-pair message exchange at decision time, reached 70.7% of optimal, making explicit the accuracy cost of decentralization; a feature ablation over local channel statistics identifies information asymmetry as a major, though not necessarily the only, contributor to this cost. Both schedulers retained more than 97% of their perfect-CSI accuracy under independent estimation errors up to $\varepsilon^{2}=0.5$, and at least 96% of it under SNR-dependent errors, stale estimates, and 3-bit quantized reporting. These results establish robustness to imprecise channel estimates, not to missing ones: when the estimates of silent peers were missing, both schedulers lost substantially more accuracy, the centralized one considerably more in relative terms, and with 30% or more silent pairs neither retained its advantage over the corresponding heuristic. The main accuracies are averages over five independently trained models, whose spread is small compared with the reported margins. Together, these results indicate that ray-traced, site-specific channel data can train practical D2D schedulers at both ends of the coordination spectrum, and they provide a quantitative basis for choosing between the two depending on the signaling budget available in a given deployment. The distributed scheduler in particular is applicable beyond conventional cellular D2D to the infrastructure-free sensor and IoT device networks envisioned for 6G [4], with the caveat that its accuracy cost relative to centralized scheduling grows with the number of contending pairs (Section 5.6).

## Figures and Tables

**Table 1:** Positioning of this work relative to representative prior studies on interference-network scheduling and power control. “Distributed comm. at inference” denotes information exchange between different links/pairs required at run time, excluding one-time model/parameter distribution and the passive overhearing of standard sidelink transmissions used for local CSI acquisition (Section 4.2). “Decision variable” denotes the quantity each method optimizes, which determines whether it addresses the same problem as this work.

Study	Channel Model	Decision Variable	Execution	Training Paradigm	Distributed Comm. at Inference
WMMSE [5]	Given CSI (any model)	Continuous power	Centralized	Iterative optimization	N/A
ITLinQ [6]	Stochastic (path loss + fading)	Binary on/off	Distributed	Rule-based (SIR threshold)	Per-slot 2-phase signaling
Sun et al. [7]	Stochastic (Rayleigh)	Continuous power	Centralized	Supervised (imitates WMMSE)	N/A
REGNN [8]	Stochastic (fading graph)	Continuous power	Centralized	Model-free RL, primal-dual	N/A
Shen et al. [9]	Stochastic (Rayleigh)	Continuous power/beamformer	Centralized	Unsupervised/RL	N/A
UWMMSE [10]	Stochastic (Rayleigh)	Continuous power	Centralized	Unsupervised (unrolled WMMSE)	N/A
Lee et al. [11]	Stochastic (path loss + shadowing); distance features	Binary on/off	Centralized	Supervised (few-shot)	N/A
Zhao et al. [3]	Abstract conflict graph (no channel model)	Binary on/off	Centralized and distributed	Model-free RL (GCN)	Neighbor exchange (distributed variant)
Wang et al. [12]	Stochastic (fading graph)	Continuous power	Distributed (*K*-hop)	Model-free RL	Per-slot *K*-hop exchange
Nasir & Guo [13]	Stochastic (path loss + fading)	Discrete power level	Distributed	Multi-agent DRL (DQN)	Per-slot neighbor CSI exchange
Li & Guo (NAAC) [14]	Stochastic	Spectrum/power assignment	Distributed (CTDE)	Multi-agent actor-critic	None claimed at execution
**This work**	**Ray-traced (Sionna, Munich)**	**Binary on/off**	**Centralized and distributed (compared)**	**REINFORCE (label-free)**	**None (0-hop, distributed variant)**

**Table 2:** Simulation parameters for the ray-traced D2D dataset.

Parameter	Value
Ray-tracing scene	Sionna munich
Carrier frequency, $f_{c}$	3 GHz
Number of D2D pairs, *N*	10
Transmit power, $P_{\mathrm{tx}}^{\left(\mathrm{dBm}\right)}$	23 dBm
Noise power, $P_{n}^{\left(\mathrm{dBm}\right)}$	−94 dBm
TX/RX antenna	short dipole, 1.76 dBi (included in H; no additional antenna gain)
Device height, *h*	1.5 m
Maximum interaction depth, $L_{\max}$	5
Maximum intra-pair distance, $d_{\max}$	50 m
Minimum intra-pair distance, $d_{\min}$	1 m (10λ)
Training/validation/test snapshots	16,000/2000/6000

**Table 3:** Sum-rate, percentage of the brute-force-optimal sum-rate, and per-sample inference latency at N=10 (6000-snapshot test set, $P_{\mathrm{tx}}^{\left(\mathrm{dBm}\right)}=23$ dBm). Learned schedulers: mean ± standard deviation over five training seeds. Latency: RTX 4090 GPU, batch 128, median of ten repetitions (five for brute force and FPLinQ; interquartile ranges and CPU values in Table 4); latency does not depend on the trained weights. LS: 1-bit-flip local search post-processing; the latency reported for a “+ LS” row is cumulative, i.e., initial schedule generation plus local-search refinement. FPLinQ uses 1000 fixed iterations with the binarization rule of its public reference implementation; the Lee et al. scheduler is the authors’ public implementation ported to this dataset (Section 5.1) and trained with five seeds.

Algorithm	Category	Sum-Rate (bps/Hz)	% of Optimal	Latency (μs/Sample)
Brute-force (optimal)	—	51.3911	100.00	869
Proposed GNN	Centralized	48.62 ± 0.06	94.60 ± 0.13	9.77
Proposed GNN + LS	Centralized	50.11 ± 0.02	97.51 ± 0.04	103
Greedy	Centralized	47.8402	93.09	34.4
Greedy + LS	Centralized	49.0412	95.43	121
FPLinQ [16]	Centralized	46.5272	90.54	2040
FPLinQ + LS	Centralized	48.9601	95.27	2070
Lee et al. graph embedding [11]	Centralized	21.75 ± 1.38	42.32 ± 2.69	7.85
Proposed distributed scheduler	Distributed	36.33 ± 0.14	70.70 ± 0.27	10.6
SIR-local (SIR > 3 dB)	Distributed	34.7058	67.53	1.30
Direct threshold (SNR > 0 dB)	Distributed	21.9194	42.65	0.930
All-on	Distributed	21.9192	42.65	0.0990
Highest-one	Reference	22.0917	42.99	0.940

**Table 4:** Per-sample inference latency in μs, median [interquartile range] over ten repetitions (five for brute force and FPLinQ), on an RTX 4090 GPU and on one thread of a 13th-generation Intel Core i9 CPU. Upper part: N=10 test set, batch 128 (per-sample values). Lower part: a single network (batch size 1) on synthetic channel matrices of *N* pairs (latency only); “one decision” is the MLP evaluation of a single pair, i.e., the computation performed by one device.

Method	GPU (RTX 4090)	CPU (1 Thread)
Brute-force	869 [868, 874]	433 [431, 454]
Proposed GNN	9.77 [9.12, 9.94]	109 [107, 115]
Proposed GNN + LS	103 [102, 105]	134 [132, 137]
Greedy	34.4 [34.2, 34.8]	8.26 [8.23, 8.33]
Greedy + LS	121 [120, 122]	32.4 [32.3, 32.6]
FPLinQ	2040 [2010, 2130]	886 [868, 890]
FPLinQ + LS	2070 [2060, 2080]	906 [888, 913]
Lee et al.	7.85 [7.71, 8.30]	13.3 [13.2, 13.5]
Proposed distributed	10.6 [9.97, 12.9]	21.3 [21.0, 21.6]
SIR-local	1.30 [1.28, 1.37]	0.886 [0.882, 0.905]
Direct threshold	0.930 [0.903, 1.03]	0.501 [0.500, 0.506]
All-on	0.0990 [0.0970, 0.106]	0.0190 [0.0190, 0.0190]
Highest-one	0.940 [0.920, 0.986]	0.653 [0.647, 0.673]
Proposed GNN, one network, N=10	1230 [1150, 1320]	454 [448, 468]
Proposed GNN, one network, N=100	1340 [1210, 1720]	9370 [9140, 9460]
Proposed distributed, one network, N=10	1310 [1210, 1390]	375 [370, 381]
Proposed distributed, one network, N=100	1250 [1220, 1290]	1440 [1420, 1460]
Distributed, one decision (one pair), N=10	171 [150, 217]	40.4 [39.9, 44.2]
Distributed, one decision (one pair), N=100	154 [151, 169]	41.0 [39.7, 45.1]

**Table 5:** Feature ablation of the distributed scheduler (% of the brute-force optimum, mean ± standard deviation over three training seeds, $P_{\mathrm{tx}}^{\left(\mathrm{dBm}\right)}=23$ dBm). Each variant uses the network and training procedure of Section 4.2 with a subset of the 19 local features; the full-feature row uses seeds 0–2 of the scheduler in Table 3. N=5: sub-sampled test networks as in Section 5.6.

Feature Set	Features	Dim.	N=10 (%)	N=5 (%)
Direct only	${\left|H\left[i,i\right]\right|}^{2}$	1	62.05 ± 0.29	63.56 ± 0.80
Direct + row	direct, 9 row statistics	10	69.46 ± 0.16	76.32 ± 0.15
Direct + column	direct, 9 column statistics	10	69.51 ± 0.22	76.67 ± 0.46
Row + column	9 row + 9 column statistics	18	70.49 ± 0.28	79.42 ± 0.36
Full (proposed)	all	19	70.87 ± 0.21	79.68 ± 0.33

**Table 6:** Generalization of the centralized GNN and distributed schedulers (both trained only at N=10) to networks of N=2,…,10 pairs, $P_{\mathrm{tx}}^{\left(\mathrm{dBm}\right)}=23$ dBm. Values other than the “Optimal” column are percentages of the brute-force-optimal sum-rate recomputed exactly at each *N*; learned schedulers: mean ± standard deviation over five training seeds. The distributed scheduler is evaluated without local-search post-processing, consistent with Table 3.

*N*	Optimal (bps/Hz)	GNN (%)	GNN + LS (%)	Distributed (%)	Greedy + LS (%)	Highest-One (%)
2	26.2197	94.41 ± 3.09	99.58 ± 0.29	85.84 ± 1.56	100.00	72.20
3	32.6552	94.96 ± 1.52	99.19 ± 0.16	85.72 ± 1.00	98.79	60.65
4	37.2056	95.40 ± 0.92	98.90 ± 0.05	80.68 ± 1.26	98.09	54.78
5	40.7645	95.63 ± 0.62	98.68 ± 0.03	79.49 ± 0.46	97.54	51.09
6	43.5226	95.48 ± 0.39	98.30 ± 0.02	76.50 ± 0.70	96.97	48.62
7	46.0086	95.36 ± 0.26	98.04 ± 0.03	75.54 ± 0.45	96.47	46.64
8	48.0522	95.21 ± 0.21	97.89 ± 0.04	73.42 ± 0.22	96.05	45.16
9	49.8724	94.95 ± 0.14	97.62 ± 0.04	72.16 ± 0.34	95.58	43.93
10	51.3911	94.60 ± 0.13	97.51 ± 0.04	70.70 ± 0.27	95.43	42.99

**Table 7:** Percentage of the brute-force-optimal sum-rate at N=10 under independent CSI error, as a function of the per-link normalized estimation error $\varepsilon^{2}$ in (12) ($P_{\mathrm{tx}}^{\left(\mathrm{dBm}\right)}=23$ dBm). All schedulers observe the same perturbed matrix $\hat{H}$; sum-rate and the brute-force optimum in the denominator are always evaluated on the true channel H, so the reference sum-rate is fixed at 51.3911 bps/Hz throughout. Learned schedulers: mean over five training seeds (standard deviation at most 0.35 percentage points).

$\varepsilon^{2}$	GNN (%)	GNN + LS (%)	Greedy (%)	Greedy + LS (%)	Distributed (%)	SIR-Local (%)
0.00	94.60	97.51	93.09	95.43	70.70	67.53
0.01	94.58	97.49	92.99	95.30	70.65	67.42
0.02	94.55	97.48	92.94	95.24	70.62	67.30
0.05	94.44	97.33	92.79	95.09	70.59	67.30
0.10	94.19	97.10	92.60	94.85	70.49	66.96
0.20	93.54	96.46	91.89	94.19	70.09	66.63
0.30	92.87	95.73	91.18	93.49	69.62	66.28
0.50	92.08	94.95	90.21	92.60	68.99	66.00

**Table 8:** Retained fraction (%) of each method’s perfect-CSI accuracy under further CSI impairments at N=10 ($P_{\mathrm{tx}}^{\left(\mathrm{dBm}\right)}=23$ dBm; learned schedulers: mean over five training seeds). *q*: fraction of silent pairs, whose cross-link row and column entries are either set to zero (missing) or replaced by stale estimates ($\varepsilon^{2}=0.5$). $G_{p}$: pilot processing gain. Quantization: uniform in dB over [−140,−40] dB. ^†^ Additional stress test with an unrealistically poor pilot.

Impairment	GNN	GNN + LS	Greedy + LS	Distributed	SIR-Local
Silent peers, missing =0, q=0.1	80.5	79.8	80.6	91.0	92.5
Silent peers, missing =0, q=0.3	61.1	59.7	61.2	76.4	80.7
Silent peers, missing =0, q=0.5	52.6	51.1	52.5	67.4	72.2
Silent peers, stale, q=0.3	98.9	98.9	99.2	99.6	99.4
Silent peers, stale, q=0.5	98.5	98.6	98.9	99.2	98.9
SNR-dependent error, $G_{p}=0$ dB	100.0	100.0	99.9	100.0	100.0
SNR-dependent error, $G_{p}=-30$ dB ^†^	98.8	98.8	98.9	99.8	99.7
Quantization, 4 bits	99.4	99.7	99.6	98.9	99.6
Quantization, 3 bits	97.4	98.4	98.1	96.1	98.7
Quantization, 2 bits	90.8	94.6	94.1	92.6	91.5

**Table 9:** Percentage of the brute-force-optimal sum-rate at N=10 versus the conducted transmit power, for schedulers trained at 23 dBm and evaluated without retraining (learned schedulers: mean over five training seeds).

$P_{\mathrm{tx}}$ (dBm)	Optimal (bps/Hz)	GNN	GNN + LS	Greedy	Greedy + LS	Distributed	SIR-Local
17	48.02	94.35	97.76	94.43	96.47	72.94	70.62
20	49.67	94.62	97.67	93.76	95.91	71.87	69.10
23	51.39	94.60	97.51	93.09	95.43	70.70	67.53
26	53.20	94.34	97.27	92.31	94.76	69.44	65.92
29	55.09	93.89	96.97	91.53	94.09	68.14	64.31

## Data Availability

The ray-traced D2D channel dataset (24,000 snapshots at N=10 over the Sionna Munich scene), the waypoint layout, the trained model weights, the dataset-generation, training, and evaluation code, and the result files underlying every table and figure are openly available in Zenodo at https://doi.org/10.5281/zenodo.22078046. The dataset is also fully regenerable from the publicly available Sionna Munich scene using the procedure and parameter values specified in Section 3.2 and Table 2.

## Abbreviations

The following abbreviations are used in this manuscript:

D2D	Device-to-device
GNN	Graph neural network
CTDE	Centralized training, decentralized execution
CSI/CSI-RS	Channel state information/CSI reference signal
DM-RS	Demodulation reference signal
PSFCH	Physical sidelink feedback channel
TDD	Time-division duplex
SINR	Signal-to-interference-plus-noise ratio
SIR	Signal-to-interference ratio
SNR	Signal-to-noise ratio
MWIS	Maximum-weight independent set
LS	Local search
LoS/NLoS	Line-of-sight/non-line-of-sight
TX/RX	Transmitter/receiver

## References

[1] Annu, Rajalakshmi P. (2024). Towards 6G V2X Sidelink: Survey of Resource Allocation—Mathematical Formulations, Challenges, and Proposed Solutions. IEEE Open J. Veh. Technol..

[2] Luo Z.Q., Zhang S. (2008). Dynamic Spectrum Management: Complexity and Duality. IEEE J. Sel. Top. Signal Process..

[3] Zhao Z., Verma G., Rao C., Swami A., Segarra S. (2023). Link Scheduling Using Graph Neural Networks. IEEE Trans. Wirel. Commun..

[4] Gismalla M.S.M., Azmi A.I., Salim M.R., Abdullah M.F.L., Iqbal F.M., Mabrouk W.A., Othman M.A.B., Ashyap A.Y.I., Mohd Supa’at A.S. (2022). Survey on Device to Device (D2D) Communication for 5GB/6G Networks: Concept, Applications, Challenges, and Future Directions. IEEE Access.

[5] Shi Q., Razaviyayn M., Luo Z.Q., He C. (2011). An Iteratively Weighted MMSE Approach to Distributed Sum-Utility Maximization for a MIMO Interfering Broadcast Channel. IEEE Trans. Signal Process..

[6] Naderializadeh N., Avestimehr A.S. (2014). ITLinQ: A New Approach for Spectrum Sharing in Device-to-Device Communication Systems. IEEE J. Sel. Areas Commun..

[7] Sun H., Chen X., Shi Q., Hong M., Fu X., Sidiropoulos N.D. (2018). Learning to Optimize: Training Deep Neural Networks for Interference Management. IEEE Trans. Signal Process..

[8] Eisen M., Ribeiro A. (2020). Optimal Wireless Resource Allocation with Random Edge Graph Neural Networks. IEEE Trans. Signal Process..

[9] Shen Y., Shi Y., Zhang J., Letaief K.B. (2021). Graph Neural Networks for Scalable Radio Resource Management: Architecture Design and Theoretical Analysis. IEEE J. Sel. Areas Commun..

[10] Chowdhury A., Verma G., Rao C., Swami A., Segarra S. (2021). Unfolding WMMSE Using Graph Neural Networks for Efficient Power Allocation. IEEE Trans. Wirel. Commun..

[11] Lee M., Yu G., Li G.Y. (2021). Graph Embedding-Based Wireless Link Scheduling with Few Training Samples. IEEE Trans. Wirel. Commun..

[12] Wang Z., Eisen M., Ribeiro A. (2022). Learning Decentralized Wireless Resource Allocations with Graph Neural Networks. IEEE Trans. Signal Process..

[13] Nasir Y.S., Guo D. (2019). Multi-Agent Deep Reinforcement Learning for Dynamic Power Allocation in Wireless Networks. IEEE J. Sel. Areas Commun..

[14] Li Z., Guo C. (2020). Multi-Agent Deep Reinforcement Learning Based Spectrum Allocation for D2D Underlay Communications. IEEE Trans. Veh. Technol..

[15] Zhao X., Lu S., Shi Q., Luo Z.Q. (2023). Rethinking WMMSE: Can Its Complexity Scale Linearly With the Number of BS Antennas?. IEEE Trans. Signal Process..

[16] Shen K., Yu W. (2017). FPLinQ: A Cooperative Spectrum Sharing Strategy for Device-to-Device Communications. Proceedings of the IEEE International Symposium on Information Theory (ISIT), Aachen, Germany, 25–30 June 2017.

[17] Wu X., Tavildar S., Shakkottai S., Richardson T., Li J., Laroia R., Jovicic A. (2013). FlashLinQ: A Synchronous Distributed Scheduler for Peer-to-Peer Ad Hoc Networks. IEEE/ACM Trans. Netw..

[18] Shan Z., Yi X., Liang L., Liao C.S., Jin S. (2025). GRLinQ: A Hybrid Model/Data-Driven Spectrum Sharing Mechanism for Device-to-Device Communications. IEEE Trans. Commun..

[19] Shen Y., Zhang J., Song S.H., Letaief K.B. (2023). Graph Neural Networks for Wireless Communications: From Theory to Practice. IEEE Trans. Wirel. Commun..

[20] Veličković P., Cucurull G., Casanova A., Romero A., Liò P., Bengio Y. Graph Attention Networks. Proceedings of the 6th International Conference on Learning Representations (ICLR).

[21] Lowe R., Wu Y., Tamar A., Harb J., Abbeel P., Mordatch I. Multi-Agent Actor-Critic for Mixed Cooperative-Competitive Environments. Proceedings of the Advances in Neural Information Processing Systems 30 (NeurIPS 2017).

[22] Williams R.J. (1992). Simple Statistical Gradient-Following Algorithms for Connectionist Reinforcement Learning. Mach. Learn..

[23] Hoydis J., Ait Aoudia F., Cammerer S., Nimier-David M., Binder N., Marcus G., Keller A. (2023). Sionna RT: Differentiable Ray Tracing for Radio Propagation Modeling. Proceedings of the 2023 IEEE Globecom Workshops (GC Wkshps), Kuala Lumpur, Malaysia, 4–8 December 2023.

[24] Hoydis J., Cammerer S., Ait Aoudia F., Vem A., Binder N., Marcus G., Keller A. (2022). Sionna: An Open-Source Library for Next-Generation Physical Layer Research. arXiv.

[25] Alkhateeb A. DeepMIMO: A Generic Deep Learning Dataset for Millimeter Wave and Massive MIMO Applications. Proceedings of the Information Theory and Applications Workshop (ITA).

[26] Pegurri R., Linsalata F., Moro E., Hoydis J., Spagnolini U. (2025). Toward Digital Network Twins: Integrating Sionna RT in ns-3 for 6G Multi-RAT Networks Simulations. Proceedings of the IEEE INFOCOM 2025—IEEE Conference on Computer Communications Workshops (INFOCOM WKSHPS), London, UK, 19 May 2025.

[27] Goldsmith A. (2005). Wireless Communications.

[28] NVIDIA Corporation Sionna Documentation. https://nvlabs.github.io/sionna/.

[29] NVIDIA Corporation Introduction to Sionna RT. https://nvlabs.github.io/sionna/rt/tutorials/Introduction.html.

[30] 3GPP. NR; Physical Channels and Modulation. Technical Specification (TS) 38.211, 3rd Generation Partnership Project (3GPP), 2024. V18.2.0. https://www.etsi.org/deliver/etsi_ts/138200_138299/138211/18.02.00_60/ts_138211v180200p.pdf.

[31] 3GPP. NR; Physical Layer Procedures for Data. Technical Specification (TS) 38.214, 3rd Generation Partnership Project (3GPP), 2024. V18.3.0. https://www.etsi.org/deliver/etsi_ts/138200_138299/138214/18.03.00_60/ts_138214v180300p.pdf.

[32] Feriani A., Hossain E. (2021). Single and Multi-Agent Deep Reinforcement Learning for AI-Enabled Wireless Networks: A Tutorial. IEEE Commun. Surv. Tutor..

[33] Kool W., van Hoof H., Welling M. Buy 4 REINFORCE Samples, Get a Baseline for Free!. Proceedings of the ICLR 2019 Workshop on Deep Reinforcement Learning Meets Structured Prediction.

[34] Truong K.T., Heath R.W. (2013). Effects of Channel Aging in Massive MIMO Systems. J. Commun. Netw..

